# *C. elegans* ageing is accelerated by a self-destructive reproductive programme

**DOI:** 10.1038/s41467-023-40088-1

**Published:** 2023-07-20

**Authors:** Carina C. Kern, Shivangi Srivastava, Marina Ezcurra, Kuei Ching Hsiung, Nancy Hui, StJohn Townsend, Dominik Maczik, Bruce Zhang, Victoria Tse, Viktoras Konstantellos, Jürg Bähler, David Gems

**Affiliations:** 1 Institute of Healthy Ageing, and Research Department of Genetics, Evolution and Environment, University College London, London, WC1E 6BT UK; 2 School of Biosciences, Stacey Building, University of Kent, Canterbury, Kent, CT2 7NJ UK; 3 Molecular Biology of Metabolism Laboratory, The Francis Crick Institute, London, NW1 1AT UK

**Keywords:** Evolutionary developmental biology, Animal physiology, Ageing

## Abstract

In post-reproductive *C. elegans*, destructive somatic biomass repurposing supports production of yolk which, it was recently shown, is vented and can serve as a foodstuff for larval progeny. This is reminiscent of the suicidal reproductive effort (reproductive death) typical of semelparous organisms such as Pacific salmon. To explore the possibility that *C. elegans* exhibits reproductive death, we have compared sibling species pairs of the genera *Caenorhabditis* and *Pristionchus* with hermaphrodites and females. We report that yolk venting and constitutive, early pathology involving major anatomical changes occur only in hermaphrodites, which are also shorter lived. Moreover, only in hermaphrodites does germline removal suppress senescent pathology and markedly increase lifespan. This is consistent with the hypothesis that *C. elegans* exhibit reproductive death that is suppressed by germline ablation. If correct, this would imply a major difference in the ageing process between *C. elegans* and most higher organisms, and potentially explain the exceptional plasticity in *C. elegans* ageing.

## Abstract (web summary)

*Caenorhabditis elegans* is used as a model species to investigate ageing, yet has a very high degree of plasticity in lifespan. This study argues that ageing in *C. elegans* is driven by suicidal reproductive effort, unlike many other organisms.

## Introduction

The mechanisms of ageing are likely to differ between organisms with semelparous and iteroparous life histories. Semelparous species exhibit only a single reproductive episode, which often involves a degree of reproductive effort that is so intense that it leads to rapid death (reproductive death)^1,2^. Organisms exhibiting semelparous reproductive death range from monocarpic plants to Pacific salmon. By contrast, iteroparous species, which include most mammals, are capable of multiple rounds of reproduction and experience a more gradual ageing process.

To be precise about the meaning of the term semelparity: although some but not all organisms with a single reproductive episode exhibit reproductive death, the term semelparity is often used to specifically denote semelparous organisms that exhibit reproductive death. Concomitantly, the occurrence of reproductive death in organisms with only a single reproductive episode is definitive of semelparity in the second sense of the term.

Since the late 1970s, the nematode *Caenorhabditis elegans* has been used intensively as a model organism to attempt to understand the causes of ageing. This led to the discovery of a remarkably high degree of plasticity in ageing in this species^3–5^ with up to 10-fold increases in lifespan having been observed^6^. *C. elegans* is also unusual in the severity and singularly early appearance of senescent pathology, particularly affecting organs involved in reproduction^7–12^. In post-reproductive *C. elegans* hermaphrodites, intestinal biomass is converted into yolk leading to intestinal atrophy and yolk accumulation^9,13^. We recently showed that post-reproductive mothers vent yolk which can function as a milk (or “yolk milk”), that is consumed by larval progeny supporting growth and reproduction^14^.

This type of self-destructive reproductive effort, where biomass repurposing promotes organ degeneration, often occurs in semelparous organisms that undergo reproductive death. Removal of the germline greatly increases lifespan in both *C. elegans* and Pacific salmon, in the latter case by suppressing reproductive death^5,15^. Taken together with its single reproductive episode, these observations raise the possibility that the marked plasticity in *C. elegans* lifespan is a function of suppression of reproductive death, i.e. that this species is semelparous.

A further hypothetical possibility is that reproductive death has evolved in *C. elegans* as a consequence of hermaphroditism. Nematode species of the genus *Caenorhabditis* are either gonochoristic, with approximately equal numbers of females and males, or androdioecious, with self-fertilising hermaphrodites and rare males^16^. Protandrous hermaphroditism (where sperm are produced first and then oocytes) allows *C. elegans* to rapidly colonise new food patches, but at the cost of a shorter reproductive span due to depletion of self-sperm^16,17^. The capacity to convert somatic biomass into yolk to feed to offspring may allow post-reproductive *C. elegans* to reduce this cost, and promote inclusive fitness^14^. If correct, this predicts that reproductive death will occur in *Caenorhabditis* hermaphrodites but not females, where obligate reproduction by mating with males supports longer reproductive spans.

In this study we use a cross-species comparative approach to explore the possibility that reproductive death occurs in *C. elegans* and is suppressed by germline ablation. That *C. elegans* might be semelparous raises interesting questions about the relevance of mechanisms of ageing identified in this species to those operative in iteroparous species (such as humans).

## Results

### Yolk venting in *Caenorhabditis* hermaphrodites but not females

**Fig. 1:**
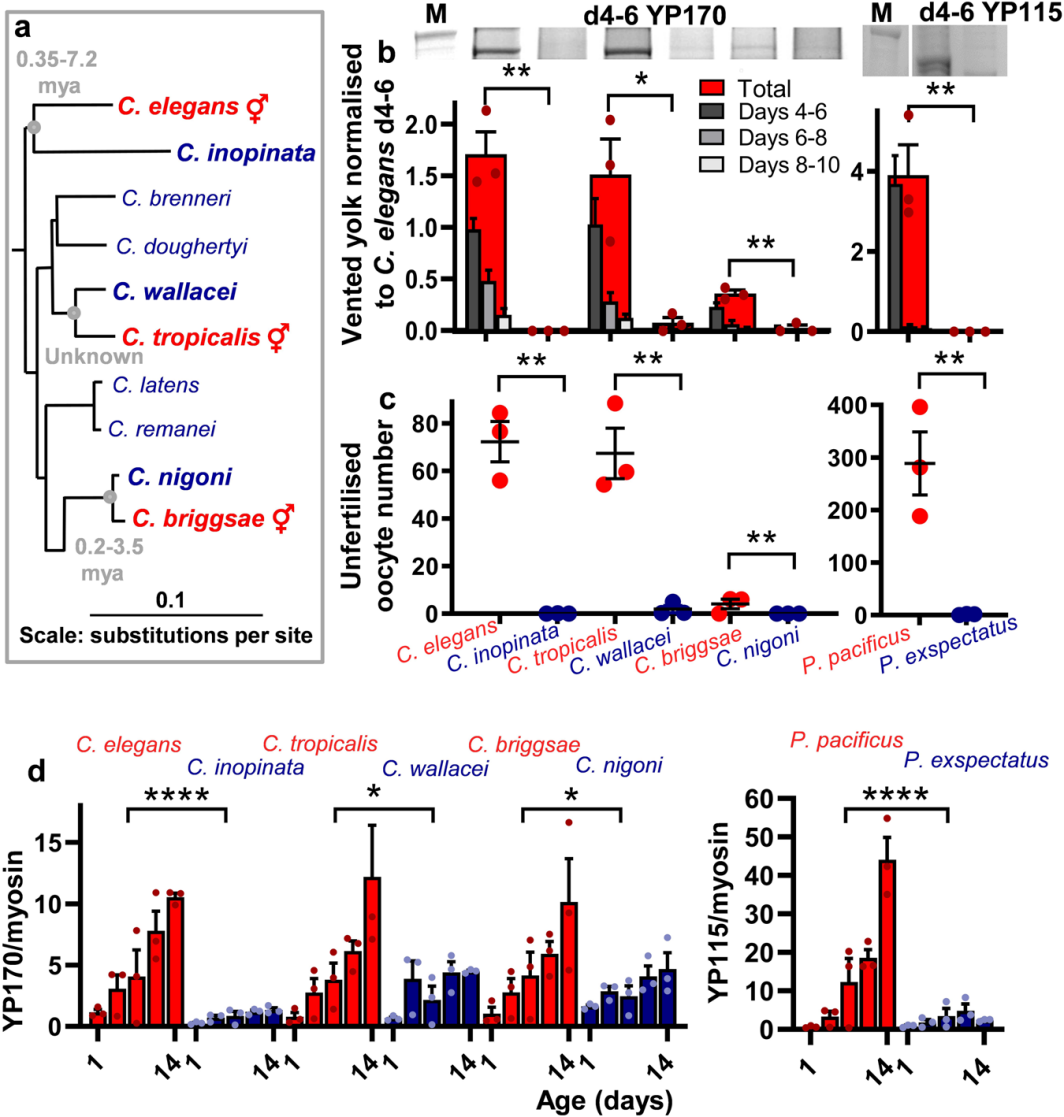
*Caenorhabditis* hermaphrodites exhibit yolk venting and a shorter lifespan. **a** Phylogenetic tree showing androdioecious and gonochoristic *Caenorhabditis* sibling species^76^. Estimate of the time since the origin of selfing in hermaphrodites: *C. elegans*, 0.35–7.2 MYA; *C. briggsae*, 0.2–3.5 MYA (ref. ^77^); *C. tropicalis*, still unknown. **b** Yolk venting present in hermaphrodites but not in females (*n* = 100 per trial). Top: major YP at the peak of venting. Bottom: Quantitative data normalised to total yolk on d4-6 in *C. elegans*. M = Marker. For full gel see Source Data. **c** Unfertilised oocytes are laid by hermaphrodites but not females (3 trials; *n* = 10 per trial). **d** Greater internal levels of major YP in *Caenorhabditis* hermaphrodites and *P. pacificus* hermaphrodites. YP bands normalised to myosin to adjust for species differences in body size. One-way ANCOVA (*n* = 10). For all internal YPs and gel image see Supplementary Fig. 1c, d. **b,** **d** Protein gel electrophoresis data with colloidal Coomassie blue staining. **b**-**e** Animals are unmated. **b,c,d** Mean ± s.e.m. of 3 trials with each dot representing one trial. **b,** **c** One-way ANOVA (Bonferroni correction) or two-tailed unpaired *t*-test. * *p* < 0.05, ** *p* < 0.01, *** *p* < 0.0001, **** *p* < 0.00001 (*p* value from left to right = **b**: 0.0014, 0.0145, 0.0012, 0.0070; **c**: 0.0010, 0.0033, 0.0080, 0.0087; **d**: 0.0000, 0.0133, 0.0133, 0.0000).

The occurrence of self-destructive biomass repurposing to support yolk production for venting in post-reproductive *C. elegans* hermaphrodites is reminiscent of mechanisms operative in organisms with reproductive death^2^. If correct, the hypothesis that reproductive death evolved after the emergence of protandry in the ancestor of *C. elegans* predicts that, among species of *Caenorhabditis*, yolk venting will occur in androdioecious but not gonochoristic species. To test this, we compared three pairs of sibling species in this genus, where one is androdioecious (A) and the other gonochoristic (G): *C. elegans* (A) vs *C. inopinata* (G), *C. briggsae* (A) vs *C. nigoni* (G), and *C. tropicalis* (A) vs *C. wallacei* (G), respectively (Fig. 1a). These pairs represent three independent occurrences of the evolution of hermaphroditism from gonochoristic ancestors^18^. For each pair, both yolk venting and copious laying of unfertilised oocytes (which act as vectors for yolk^14^) was seen in hermaphrodites but not females (Fig. 1b, c; Supplementary Fig. 1a, b). As in *C. elegans*, vitellogenin (yolk protein) accumulation continued into later life to high levels in *C. briggsae* and *C. tropicalis* hermaphrodites, but not in *C. inopinata*, *C. nigoni* and *C. wallacei* females (Fig. 1d). We also compared an androdioecious-gonochoristic sibling species pair from the free-living nematode genus *Pristionchus* and, again, only the former vented yolk and oocytes, and accumulated vitellogenin internally in later life (Fig. 1b–d; Supplementary Fig. 1a–d). *Pristionchus* proved to lack the larger vitellogenin species corresponding to YP170 in *Caenorhabditis*; instead, accumulation of only the smaller species equivalent to *Caenorhabditis* YP115/YP88 was seen (Supplementary Fig. 1c, d).

### Hermaphrodites are shorter lived than females

The hypothesis that yolk venting is indicative of semelparity, together with the presence of yolk venting in hermaphrodites but not females, predicts that only the former will exhibit reproductive death, i.e. that females will be longer lived and show greatly reduced pathology relative to hermaphrodites. Consistent with this, lifespan was longer in females than in hermaphrodites for the *C. tropicalis* (A)/*C. wallacei* (G) and *C. briggsae* (A)/*C. nigoni* (G) pairs but, as previously reported^19^, this was not the case for the *C. elegans* (A)/*C. inopinata* (G) pair (Supplementary Fig. 2a, b).

**Fig. 2:**
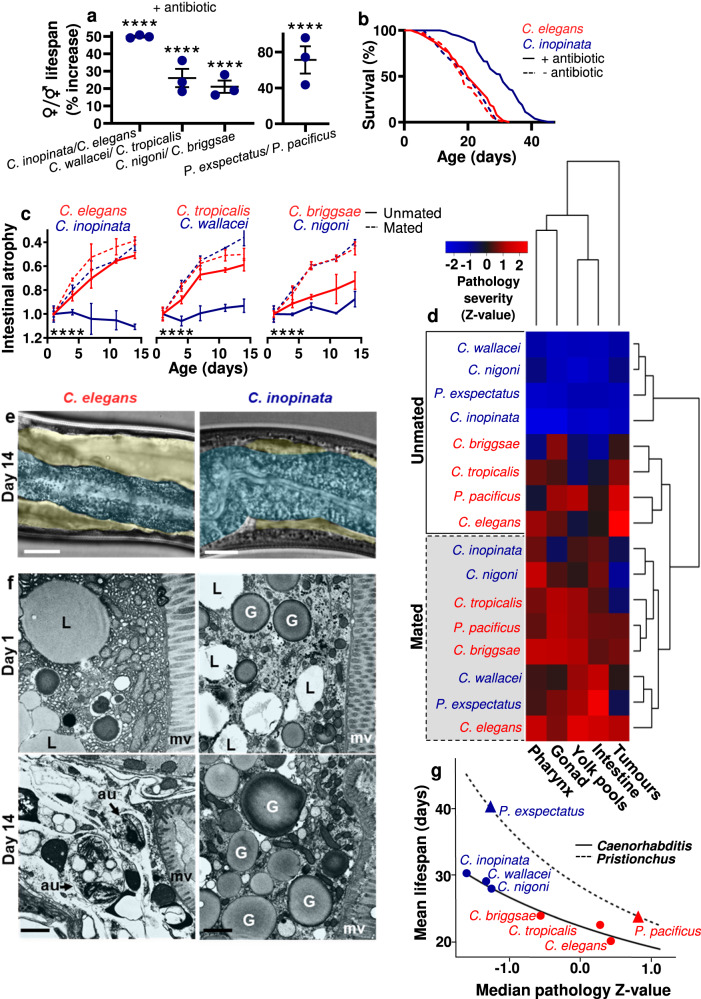
Mated females exhibit pathology similar to unmated hermaphrodites. **a** Females are longer lived than sibling species hermaphrodites (unmated, with antibiotic). Mean ± s.e.m. of 3 trials. Log-rank (Mantel-Cox) test. **b** Female *C. inopinata* are longer-lived than *C. elegans* when bacterial infection is prevented using an antibiotic (carbenicillin) (this figure; combined data of 3 trials; *n* = 100 per trial) or UV irradiation of *E. coli* (Supplementary Fig. 2d). **a**, **b** For statistical details, see Supplementary Table 1, and for raw lifespan, data see Source Data File. **c** Intestinal atrophy is constitutive in hermaphrodites and mating-induced in females. Mean ± s.e.m. of 3 trials (*n* = 10 per trial per time point). One-way ANCOVA with score normalised to d1 to determine the change in percentage of intestinal mass. Trial terms were not statistically significant across all pathologies and, therefore, trial was not considered further a variable. For details of statistic tests see the methods section. For measurement of pathologies, statistical comparisons between species and treatments, and representative images of pathologies used for scoring, see Supplementary Fig. 4. **d** Reproductive death-associated pathologies are constitutive in hermaphrodites and induced in females by mating. The heat map compares differences in pathology progression across species and treatments by transforming calculated gradients from pathology measurements through time into Z-scores (c.f. Supplementary Fig. 4a, d; 3 trials, where *n* = 10 per time point per trial, with 5 time points up to d14, when pathology peaks for *C. elegans*). This allows comparison between different pathologies by normalising levels of a given pathology to the average level of that pathology in the group (represented by a Z-score of 0, with a Z-score of +2 representing the maximum pathology severity in the group and −2 the healthiest animals). Hierarchical clustering is based on pair-wise Euclidean distances. **e** Intestinal atrophy (blue) and yolk pool accumulation (yellow) in ageing *C. elegans* but not *C. inopinata* (d14, unmated). Representative Nomarski images. Scale 25 µm. **f** Severe degeneration of intestinal ultrastructure in ageing *C. elegans* but not *C. inopinata* (day 14, unmated). Note loss of organelles and ground substance. Representative TEM cross-section images through the intestine. mv, microvilli; au, autophagosome-like structure, L, lipid droplets; G, yolk or gut granules (tentative identification). Scale 1 µm (20,000x). **g** Correspondence between pathology severity and lifespan. Linear regression of the median of all pathology Z-scores (c.f. Fig. 2d) with lifespan. Y axis shows mean lifespan of each species (antibiotic treated). A line of best fit is drawn to connect points for the *Caenorhabditis* species; based on this a hypothetical line is drawn for the *Pristionchus* species. For regression of individual pathologies against lifespan, see Supplementary Fig. 2e. **** *p* < 0.00001.

To exclude possible species differences in susceptibility to infection by the *E. coli* food source^20^, lifespan was measured in the presence of an antibiotic (carbenicillin), and here females were longer lived in all three sibling species pairs (Fig. 2a, b; Supplementary Fig. 2a, b); this result suggests that *C. inopinata* are hyper-susceptible to bacterial infection, perhaps reflecting their distinct natural environment (syconia of fig trees). The optimal culture temperature for *C. inopinata* is higher (25–29 °C) than that for *C. elegans* (20–22 °C)^21^, raising the possibility that culture of *C. inopinata* at the sub-optimally low temperature of 20 °C might cause a disproportionately low rate of living, thereby increasing lifespan relative to *C. elegans*; however, *C. inopinata* was also longer lived than *C. elegans* at 25 °C (Fig. Supplementary 2c), arguing against this possibility. Similarly, in the *Pristionchus* sibling species pair, the hermaphrodites were shorter lived (Fig. 2a, Supplementary Fig. 2a, b), consistent with a previous report of greater longevity in *Pristionchus* females^22^.

### Severe senescent pathology in hermaphrodites but not females

Next we compared patterns of senescent pathology in the three sibling *Caenorhabditis* species pairs using Nomarski microscopy and transmission electron microscopy. Early, severe senescent pathologies in *C. elegans* adult hermaphrodites include prominent uterine tumors, gonadal atrophy and fragmentation, pharyngeal deterioration, as well as intestinal atrophy coupled with yolk accumulation^7–9,23–25^. Senescent pathologies seen in *C. elegans* were also seen in the other two hermaphroditic species of *Caenorhabditis*, but were largely absent from females, and this was true also of the *Pristionchus* sibling species pair (Fig. 2c–e). Striking degeneration of intestinal ultrastructure was seen in older *Caenorhabditis* hermaphrodites, consistent with earlier observations of *C. elegans*^7,26^ and *C. briggsae*^27^, including prominent autophagosomes (Fig. 2f; Supplementary Fig. 3). Such changes were not seen in females. Thus, females are longer lived and free of the major anatomical changes characteristic of senescent hermaphrodites.

### More severe senescent pathology is associated with shorter lifespan

The severity of early senescent pathology in hermaphrodites varied between species, with the ranking *C. elegans* > *C. tropicalis* > *C. briggsae* (Fig. 2c, d), and hermaphrodite longevity showed the inverse ranking (Supplementary Table 1). The graded variation of pathology level with lifespan is consistent with possible existence of a continuum between presence and absence of reproductive death (i.e. between semelparity and iteroparity; see below) (Fig. 2g). This could imply that *C. elegans* and *C. briggsae* represent late and early stages, respectively, in the evolution of constitutive reproductive death. Notably, the estimated time since the origin of selfing in hermaphrodites is longer ago for *C. elegans* (0.35–7.2 MYA) than for *C. briggsae* (0.2–3.5 MYA). Moreover, *C. briggsae*/*C. nigoni* inter-species mating can produce offspring^28^, consistent with relatively recent divergence of these two species from a common ancestor.

### Mated females exhibit pathology similar to unmated hermaphrodites

**Fig. 3:**
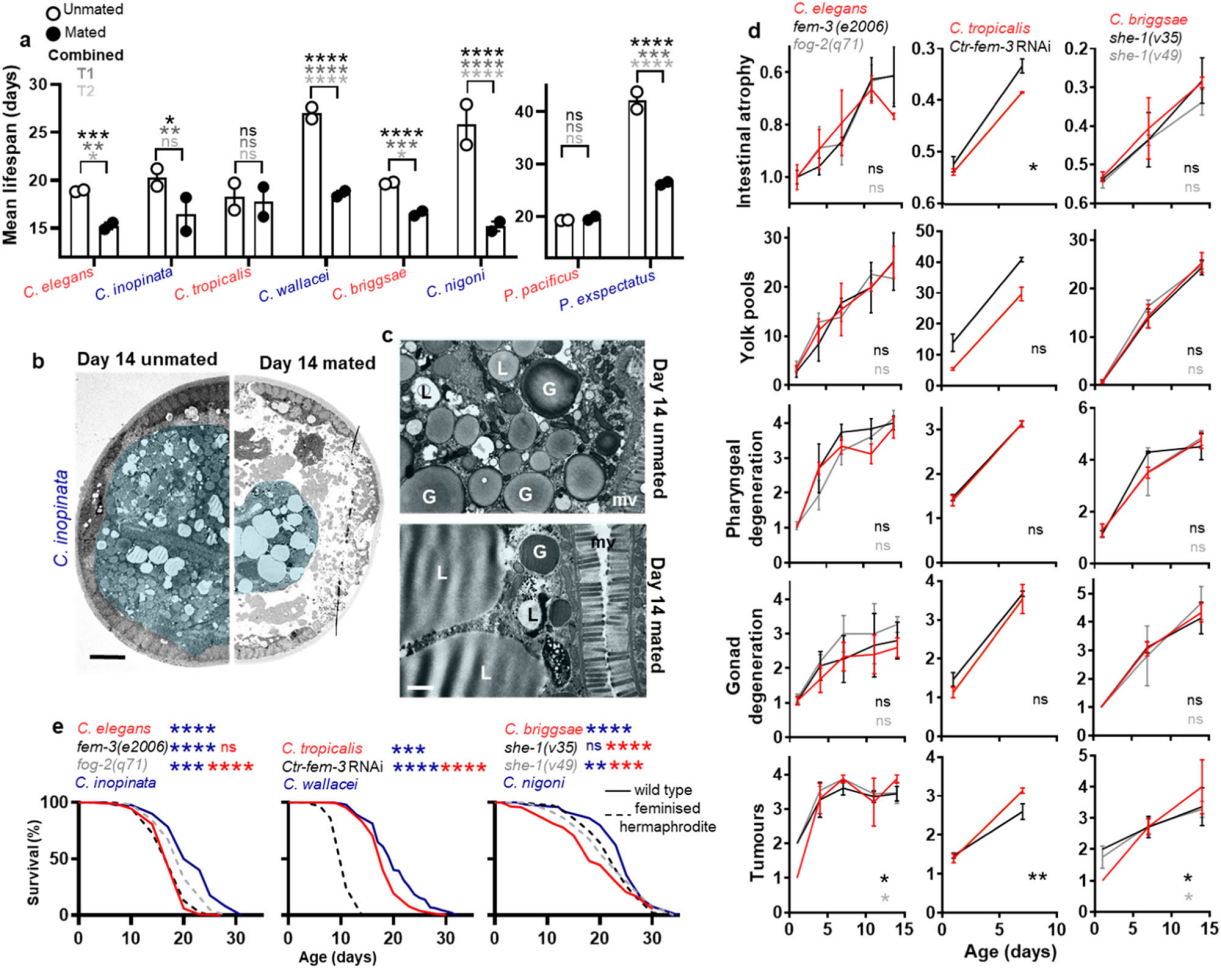
Mating induces reproductive death and reduces lifespan in females. **a** Mating in females significantly reduces lifespan, as previously shown for *C. elegans*^30,31,41^. Mean ± s.d. of 2 trials. Log-rank between mated and unmated animals. For statistical details, see Supplementary Table 2, and for raw lifespan data see Source Data File. **b** Representative TEM cross-section showing a reduction in intestinal size in *C. inopinata* upon mating. Scale 5 μm (5,000x). **c** Representative high magnification TEM images showing degenerative ultrastructural changes in the intestine in *C. inopinata* upon mating, with loss of ground structure similar to unmated *C. elegans*, and in contrast to unmated *C. inopinata*, observed (c.f. Fig. 2f). Scale 1 μm (20,000x). **d** No effect of self-sperm on senescent pathology in *Caenorhabditis* hermaphrodites in most cases. The main exception is uterine tumours which appear sooner due to earlier appearance of unfertilised oocytes in the uterus, but then develop more slowly, as previously described for *C. elegans*^24^. Also, in *C. tropicalis* a slight increase in intestinal atrophy is observed. Trials were performed at 25 °C because RNAi induced sterility in *C. tropicalis* at this temperature. Mean ± s.e.m. of 3 trials (*n* = 5–10 per time point in each trial). Cumulative Link Model with gamma link function for pharyngeal degeneration, gonadal degeneration and uterine tumours which are scored ordinals. One-way ANCOVA with score normalised to d1 to determine change in percentage of intestinal mass, and one-way ANCOVA with no normalisation for yolk pools. Trial terms were not statistically significant across all pathologies and, therefore, trial was not considered further a variable. For details of statistic tests see methods section. **e** Presence of self-sperm weakly reduces lifespan in some contexts. Trials conducted at 25 °C (see **d**) and with carbenicillin included to avoid possible confounding effects of species differences in susceptibility to bacterial infection. In previous studies of *C. elegans fog-2(q71)* females were found to be long lived in one case (25 °C, live *E. coli*)^19^ and short lived in another (20 °C, live *E. coli*)^20^; the reason for the discrepancy is unclear. Combined data of 3 trials. Statistical test (log-rank), comparison to wild-type females: blue stars; comparison to wild-type hermaphrodites: red stars. For survival curves for all trials see Supplementary Fig. 5c, for statistical details see Supplementary Table 3, and for raw lifespan data see Source Data File. * *p* < 0.05, ** *p* < 0.01, *** *p* < 0.0001, **** *p* < 0.00001.

The above comparisons were performed using unmated animals, meaning that hermaphrodites produced progeny but females did not. Thus, the differences observed could in principle reflect the presence/absence of progeny production, though it should be noted that prevention of self-progeny production per se does not affect lifespan in *C. elegans*^3,4,29^. To investigate this possibility, we compared lifespan and senescent pathology in mated animals of the different species. Mating (including exposure to male pheromones as well as copulation) shortened lifespan in most species, as previously seen in *C. elegans*^30,31^, and abrogated the greater longevity of females (Fig. 3a; Supplementary Fig. 5a, b). Mating also induced intestinal atrophy in females and enhanced it in hermaphrodites, and mated animals of all six species showed similar levels of intestinal atrophy (Figs. 2c; 3b, c). A comparable effect of mating was also seen on a range of other pathologies, where similar levels of deterioration were seen in mated females and hermaphrodites, as shown by cluster analysis of quantified pathology severity, and *Pristionchus* species showed the same trend (Fig. 2d).

These findings could imply that reproductive death is induced by mating in females. This would provide a possible explanation for how such similar patterns of pathogenesis evolved independently in the three androdioecious species: as the consequence of a switch from mating-induced reproductive death in females to constitutive reproductive death in hermaphrodites, whether or not (in the latter) reproduction by self-fertilisation has taken place. If mating induces reproductive death in females, one might expect that it would also induce yolk venting: critically, for reproductive death to be occurring, in line with semelparity, it must involve some form of fitness benefit. However, only very low levels of vented yolk were detected in mated females (Supplementary Fig. 1a). In *C. elegans* mating reduced vented yolk levels, as it did in all hermaphroditic species, likely due to increased uptake of yolk into oocytes that are subsequently fertilised. These results could imply that in females reproductive death is coupled to yolk production for egg provisioning only, but the existence of such a benefit remains unproven; thus, it remains unclear whether mating-induced pathology in females is coupled to a reproductive benefit, i.e. whether mated females exhibit reproductive death.

According to this model, the evolution of reproductive death in hermaphrodites requires constitutive activation of processes that normally require mating to be induced. The evolution of hermaphroditism in *C. elegans* likely began when females developed the capacity to generate and activate self sperm^32^. One possibility is that the emergence of self-sperm was sufficient for reproductive death to become constitutive. To investigate this we tested whether abrogating self-sperm production in *Caenorhabditis* hermaphrodites would suppress senescent pathology and extend lifespan. Interventions used were *fog-2(q71)* and *fem-3(e2006)* in *C. elegans*^33,34^, *she-1(v35)* and *she-1(v49)* in *C. briggsae*^35^, and RNAi of a putative *fem-3* orthologue (Csp11.Scaffold629.g10847, *Ctr-fem-3*) in *C. tropicalis*.

Blocking self-sperm production did not suppress the development of senescent pathology (Fig. 3d). However, it did increase lifespan in several instances - in *C. elegans* with *fog-2* but not *fem-3*, consistent in both cases with earlier findings^4,19^, and in *C. briggsae* with *she-1* - but in no case to the extent of sibling species female longevity (Fig. 3e). In *C. tropicalis Ctr-fem-3* RNAi strongly *reduced* lifespan, possibly reflecting pleiotropic effects. These results imply that constitutive reproductive death is not a simple consequence of the evolution of self-fertilisation. They do, however, leave open the possibility that the appearance of self-sperm contributes in a multi-step evolutionary process. The possibility that reproductive death in self-fertilising hermaphrodites is a consequence of reproduction itself was ruled out by the early observation that sterile hermaphrodites (e.g. due to fertilisation-defective sperm) are not long-lived^3,29^. Thus, while reproduction per se does not shorten lifespan, the presence of self-sperm might to an extent.

### Major impact of germline ablation on ageing in hermaphrodites but not females

In semelparous species where reproductive death is triggered upon reproductive maturity, pre-empting reproduction can greatly increase lifespan^1,2^. For example, removal of flowers prior to pollination can increase lifespan in soybean from 119 to 179 days^36^, and gonadectomy of Pacific salmon before spawning can increase maximum lifespan from 4 years to up to 8 years^15^. Similarly, prevention of germline development in *C. elegans* hermaphrodites greatly increases lifespan^5^ and suppresses intestinal atrophy^9^.

**Fig. 4:**
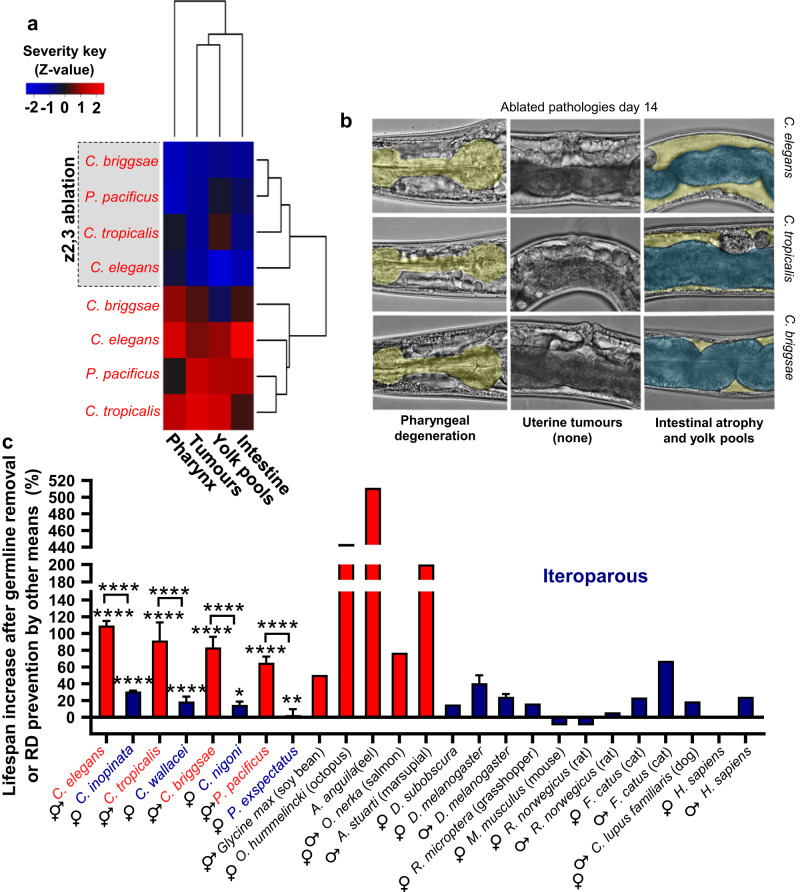
Germline ablation extends hermaphrodite lifespan by suppressing reproductive death. **a** Germline ablation strongly suppresses pathology progression in hermaphrodites (c.f. Fig. 2d; *n* = 5–10 per time point). For full pathology measurements see Supplementary Fig. 6a, and for pathology progression in germline-deficient mutants see Supplementary Fig. 6b. **b** Representative Nomarski microscopy images taken for the purpose of scoring pathologies. **c** Germline ablation strongly increases lifespan in hermaphrodites but not female nematode species. Similarly, blocking reproductive death in semelparous species strongly increases lifespan, while gonadectomy in iteroparous species overall does not. For *Caenorhabditis* and *Pristionchus* species, mean ± s.e.m. of 3 trials is shown (stars, log-rank test for ablated *vs* control animals, and Cox proportional hazard test to compare effect in hermaphrodites *vs* females). Values for other species are taken from a recent review^2^; for multiple studies of the same species, only references with both male and female data are included, and for *A. anguila* (eel) data from the most recent source^78^. For data on pathology after ablation of the entire gonad see Supplementary Fig. 6c. For individual trials, details on statistics and raw lifespan data, see Supplementary Fig. 7, Supplementary Table 4 and Source Data File, respectively. * *p* < 0.05, ** *p* < 0.01, **** *p* < 0.00001.

One possibility is that germline ablation extends *C. elegans* lifespan by suppressing reproductive death. Given the absence of putative reproductive death in unmated *Caenorhabditis* and *Pristionchus* females, this predicts that germline ablation will increase lifespan more in hermaphrodites than in females, and this proved to be the case. Germline ablation using laser microsurgery^5^ suppressed major pathologies (Fig. 4a, b) and caused large increases in lifespan in hermaphrodites, but only marginal increases in lifespan in females (Fig. 4c). Ablation of the entire *C. elegans* gonad does not suppress intestinal atrophy (Supplementary Fig. 6c). This implies that germline ablation effects on senescent pathology require the presence of the somatic gonad, as is the case for effects on lifespan^5^. This suggests that reproductive death is suppressed by signals from the somatic gonad; notably, life-extending effects of the somatic gonad involves signalling to the DAF-12 steroid hormone receptor^5,37^.

We also surveyed published reports that have assessed effects on lifespan of gonadectomy or, for some semelparous species, prevention of reproductive death by other means. This confirmed that large increases in lifespan are typical of semelparous but not iteroparous organisms (Fig. 3c). Hermaphrodite and female lifespans of germline-ablated animals in each sibling species pair were similar (Supplementary Fig. 7), supporting the hypothesis that the shorter lifespan of hermaphrodites is attributable to reproductive death.

## Discussion

Results presented in this study, taken together with earlier findings, show that a number of properties of *C. elegans* fit the criteria used to define reproductive death in semelparous species, i.e. provide support for the hypothesis that it exhibits reproductive death. *C. elegans* hermaphrodites exhibit major anatomical changes of a clearly pathological nature (degeneration, tumours) in organs linked to reproduction relatively early in adulthood, some of which result from biomass repurposing for yolk production^2,9,14,38^, while none of this is seen in unmated females. Hermaphrodites are shorter-lived than females of their sister species, but not after germline removal, which causes large increases in lifespan only in hermaphrodites. This is consistent with the hypothesis that germline removal, like gonadectomy in Pacific salmon, increases lifespan by preventing reproductive death.

In line with this, *C. elegans* males resemble *Caenorhabditis* females in not exhibiting major degenerative pathologies such as intestinal atrophy and gonad disintegration^9,23^, and germline removal in wild-type *C. elegans* males has little effect on lifespan^39^. Moreover, prevention of reproductive maturity by gonadectomy or other means typically causes large increases in lifespan in semelparous organisms but not iteroparous organisms^2,38^ (Fig. 4c).

A characteristic feature of semelparous organisms that exhibit reproductive death is that their lifespan after reproduction is very short. While in relative terms (i.e. as a proportion of their total lifespan), selfing hermaphrodites have a long post-reproductive lifespan, in absolute terms this span is very short (~2 weeks, under standard culture conditions). Moreover, sperm-depleted hermaphrodites are not necessarily post-reproductive given the occurrence of yolk venting^14^.

Mating with males induces pathological changes in females similar to those seen in unfertilised hermaphrodites (Figs. 2c, d; 3b, c), suggesting the possibility that reproductive death also occurs in mated females. However, while in hermaphrodites there is evidence that intestinal degeneration is coupled with the production of vented yolk in a fitness trade-off^9,14^, evidence for a similar trade-off in mated females is currently lacking; mating does not induce yolk venting (Supplementary Fig. 1). One possible candidate for such a benefit is increased yolk production for oocyte provisioning.

That life-shortening effects of exposure to males are promoted by both seminal fluid^31^ and male pheromone^40,41^ has been interpreted as indicative of sexual conflict^42^. Another possibility is that the life-shortening effects of males on females/hermaphrodites is a cost linked to a mutually beneficial increase in fitness arising from reproductive death. These two possibilities are not mutually exclusive; notably, the presence of self-sperm protects against the life-shortening effects of male exposure^43,44^, consistent with sexual conflict. This protective mechanism is mediated by several signalling components, including the CEH-18 transcription factor, the VAB-1 ephrin receptor^43^, insulin-like peptides and the mTOR-TFEB pathway^44^.

The occurrence of reproductive death (i.e. semelparity) in *C. elegans* would have profound implications both in terms of understanding the biology of ageing, and what one can learn from *C. elegans* as a model for ageing in general. It was previously proposed that some senescent pathologies are the result of a vitellogenic quasi-programme^45,46^, i.e. futile run-on of a yolk production programme after reproduction^7,9,13^. While this is still possible, if later yolk production provides a fitness benefit then such senescent pathologies would instead represent reproductive costs. Such benefits could accrue from yolk feeding by larvae either when they hatch inside the mother, or when external larvae consume vented yolk (indicating a cost of “lactation”)^14^. However, fitness-promoting processes to which uterine tumour formation is coupled have not (yet) been identified; thus, like the ovarian teratomas that they resemble^24^, uterine tumours do appear to be the result of quasi-programmes. Thus, both costly programmes^46^ and quasi-programmes are likely operative in *C. elegans* hermaphrodite senescence.

As specified in the introduction, the word semelparous has two overlapping meanings: (i) having only a single bout of reproduction, and (ii) reproducing once followed by reproductive death. Regarding the first sense: arguably, the distinction between semelparity and iteroparity can be difficult to apply to organisms with very short lifespans, such as *Caenorhabditis* species^47^. However, the very brief reproductive span of *C. elegans* combined with the evidence of reproductive death presented here is evidence of semelparity in the second sense of the word and, by extension, the first.

The most exciting thing about the discovery of interventions producing large increases in lifespan in *C. elegans* was the implied existence of mechanisms with powerful effects on ageing as a whole. Combined with the belief that ageing rate is a function of somatic maintenance^48^, this suggested that similar plasticity might exist in humans, affecting the entire ageing process, and provide a possible target for future interventions to greatly decelerate ageing. Here we present evidence that lifespan in *C. elegans* is limited by reproductive death. We have also argued elsewhere that reproductive death is permissive for the evolution of the rare phenomenon of programmed adaptive death, which further shortens lifespan, and that this may have occurred in *C. elegans*^49–51^. Suppression of such programmatic aetiologies of ageing provides a potential explanation for the unusually large magnitude of increases in lifespan seen in *C. elegans*. This raises the possibility that in iteroparous organisms no similar core mechanism of ageing as a whole exists that is amenable to manipulation to produce dramatic deceleration of ageing. Sadly, our findings might, in certain respects, explain away the mystique of *C. elegans* lifespan plasticity.

Would the occurrence of reproductive death mean that *C. elegans* die from mechanisms unrelated to those operative in most other organisms? We believe not. In the past, a sharp distinction was drawn between true ageing, caused by stochastic damage accumulation, and programmed ageing as observed in plants (e.g. leaf senescence) and semelparous species such as Pacific salmon^1,2^. However, it has been recognised that evolutionarily conserved regulators of phenotypic plasticity in ageing, such as the insulin/IGF-1 and mechanistic target of rapamycin (mTOR) pathways, can act through programmatic mechanisms to promote senescent pathology^45,52–54^. Inhibiting these pathways can cause large increases in *C. elegans* lifespan but also smaller effects in higher animals; for example, mutation of phosphatidylinositol 3-kinase can increase median lifespan by up to ~10-fold in *C. elegans*, but only ~1.07-fold and ~1.02-fold in *Drosophila* and mouse, respectively^6,55,56^.

**Fig. 5:**
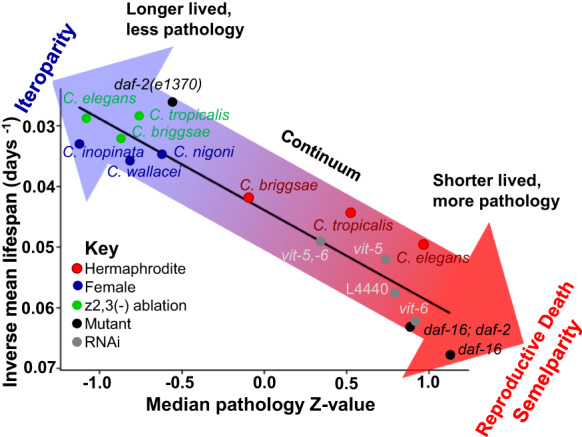
A continuum between semelparity and iteroparity. Correlation between reproductive death-associated pathologies and lifespan in different species and following different treatments in *C. elegans*, supporting the presence of a semelparity-iteroparity continuum. X axis, median pathology Z-score. Y axis, mean lifespan (unmated, antibiotic treated). Other data sources: RNAi pathology and lifespan^13^; lifespan of mutants^79^. For mutant pathology measurements, see Supplementary Fig. 8.

Given the evolutionary conservation of the role of these pathways in ageing, we suggest that mechanisms of reproductive death evolve by repurposing programmatic mechanisms that affect ageing to a small extent in iteroparous organisms^2,38^. It has been argued that semelparous and iteroparous life histories are not isolated phenomena but rather represent a continuum^57^. Supporting this is the observed gradient between the presence and absence of putative reproductive death across nematode species, and in *C. elegans* across interventions that suppress pathologies of reproductive death and extend lifespan, including germline ablation and reduced IIS (Fig. 5). Thus, even if *C. elegans* does undergo reproductive death, it remains a good model for understanding programmatic mechanisms of ageing contributing to senescent multimorbidity that are universal across metazoan organisms^46^.

## Methods

No statistical methods were used to predetermine the sample size. The experiments were not randomised. The investigators were not blinded to allocation during experiments and outcome assessment unless otherwise stated. All statistical tests were performed on raw data using GraphPad Prism 8.0 unless otherwise stated.

### Nematode culture methods and strains

Maintenance of *C. elegans* and other species was performed using standard protocols^58^. Unless otherwise stated, all strains and species were grown at 20 °C on nematode growth media (NGM) with plates seeded with *E. coli* OP50 to provide a food source. For *C. elegans*, a N2 hermaphrodite stock recently obtained from the Caenorhabditis Genetics Center was used as wild type (N2H) (ref. ^59^). Genotypes of *C. elegans* mutant strains used are described in Wormbase (www.wormbase.org), and included CB3844 *fem-3(e2006)*, CB4037 *glp-1(e2141)*, GA14 *fog-2(q71)*, GA114 *daf-16(mgDf50)*; *daf-2(e1370)*, GA1928 *daf-2(e1370)*, GR1307 *daf-16(mgDf50)* and SS104 *glp-4(bn2)*. Unless otherwise stated, strains for other nematode species were VT847 *C. briggsae*, NK74SC *C. inopinata*, JU1325 *C. nigoni*, JU1373 *C. tropicalis*, JU1873 *C. wallacei*, RS5522 *P. exspectatus* and RS2333 *P. pacificus*. *C. briggsae* female mutants RE665 *she-1(v35)* and RE770 *she-1(v49)* were also used, and compared to *C. briggsae* (AF16) from which these mutants are derived^35^. *C. inopinata* were raised at 25 °C until the L4 stage and then transferred to 20 °C since at the latter temperature they exhibit a very slow development rate^21^. Temperature-sensitive mutants (* glp-1*, *glp-4, she-1*) and accompanying controls were raised at 15 °C until the L4 stage and then shifted to 25 °C, the non-permissive temperature.

### RNAi by injection to feminise *C. tropicalis* hermaphrodites

Identification of *C. tropicalis* orthologues of *C. elegans fog-2* and *fem-3* was performed using Wormbase ParaSite^60,61^. Following protein and DNA sequence alignment^62^, *C. tropicalis* Csp11.Scaffold629.g10847 was designated as the *C. elegans fem-3* orthologue. No orthologue of *C. elegans fog-2* was found in *C. tropicalis*, which is consistent with differences in the regulation of sperm function in these two species^63^. Three RNAi targeting regions were designed to enhance the efficiency of knockdown. In order to synthesis dsRNA for microinjection, three PCR products for in vitro transcription were generated with specific primers with T7 promoter sequences (TAATACGACTCACTATAGGG) included at the 5’ ends. PCR cycle conditions were 95 °C 30 sec, 47 °C 30 sec, 72 °C 60 sec for 30 cycles. PCR products were purified through gel extraction using a QIAquick Gel Extraction Kit (QIAGEN). dsRNAs were synthesised in vitro using a MAXIscript T7 Transcription Kit (ThermoFisher), and this was followed by RNA gel electrophoresis to analyse product size and quality. Next, the three dsRNAs were mixed at a ratio of 1:1:1, at a final concentration of 0.1 μg/μl, and then injected into both arms of the gonad of a 1-day-old adult *C. tropicalis*^64^. Injected nematodes were recovered and allowed to lay eggs for 6 hr at 25 °C to obtain developmentally synchronised progeny. These were then transferred individually to wells of a 24 well plate with NGM seeded with OP50 and grown to adulthood. Approximately 50% proved to be sterile and otherwise of a normal size and appearance and were used for experimental trials. Progeny of non-injected *C. tropicalis* hermaphrodites was used as negative controls. See also Supplementary Fig. 9 and Supplementary Tables 5–7.

### Nomarski microscopy

Live nematodes were placed on 2% agar pads, and anaesthetised with a drop of 0.2% levamisole. Images were captured using either a Zeiss Axioskop 2 plus microscope with a Hamamatsu ORCA-ER digital camera C4742-95 and Velocity 6.3 software (Macintosh version) for image acquisition; or an ApoTome.2 Zeiss microscope with a Hamamatsu digital camera C13440 ORCA-Flash4.0 V3 and Zen software. Brightness and contrast were adjusted equally across the entire image, and where applicable applied equally to controls.

### Transmission electron microscopy

Nematode sample preparation was based on standard protocol^65^ (protocol 8). Briefly, animals were washed twice in M9 buffer, placed in fixing solution (2.5% glutaraldehyde, 1% paraformaldehyde in 0.1 M sucrose, 0.05 M cacodylate) and, to increase staining dye penetration, cut below the pharynx to remove the heads using a 30 G hypodermic needle. They were then rinsed 3 times in 0.2 M cacodylate, fixed in 0.5% OsO_4_ and 0.5% KFe(CN)_6_ in 0.1 M cacodylate on ice and sequentially washed in 0.1 M cacodylate and 0.1 M sodium acetate. Nematodes were then stained in 1% uranyl acetate in 0.1 M sodium acetate (pH 5.2) for 60 min, rinsed 3 times in 0.1 M sodium acetate and then rinsed overnight in Milli-Q water. Samples were then embedded in 3% seaplaque agarose, dehydrated and infiltrated using ethanol and propylene-resin series and then cured at 60 °C for 3 days.

Serial 1 μm sections were taken for light microscopy, and, from a region posterior to the posterior uterus, ultra-thin sections (70–80 nm) were cut using a diamond knife on a Reichert ultramicrotome. Comparative low-magnification images showing halves of nematode sibling species pairs were taken from approximately the same region of the animals containing the posterior intestine. Sections were collected on formvar-coated 2×1 mm slot grids which allowed low magnification images to be taken without grid bars and stained with lead citrate before being viewed and imaged in a Joel 1010 transmission electron microscope. Images were recorded using a Gatan Orius CCD camera, and obtained using Gatan imaging software. Brightness and contrast were adjusted equally across the entire image, and where applicable applied equally to controls.

### Lifespan measurements

Animals were maintained at a density of 25–30 per plate from the egg stage to minimise the population density-associated effects on ageing that occur in *C. elegans*^66^, and without the addition of FUDR. Nematode cohorts were scored every 2–3 days for the dead, and all animals transferred daily during the reproductive period, and every 6–7 days thereafter. Worms were scored as alive if they were motile or responded at all to gentle touch with a platinum worm-pick. For survival assays on UV-irradiated bacteria, 80 μl of *E. coli* OP50 was added to each NGM plate and left overnight at 20 °C. Plates were then exposed to UV for 30 min in a Stratalinker UV oven. Nematodes were raised on UV-treated bacteria from aseptic eggs. Raw mortality data for all trials is provided in Ziehm tables^67^ in the Source Data File to enable close scrutiny of data (including future reanalysis). Survival plots show combined lifespan data and individual trials, with the L4 stage of development taken as day 0. Animals which disappeared from the plate, were accidently killed during handling, died due to desiccation on the Petri dish wall, ruptured through the vulva or died due to internal hatching of larvae were recorded as censored. Censored values were taken into account in statistical analysis. Tests for statistically significant differences between the survival of cohorts employed the log-rank test, and for significant differences in the magnitude of effects of two treatments Cox proportional hazard analysis, in both cases performed using JMP software, version 14.0 (SAS Institute, Inc.).

### Counts of numbers unfertilised oocyte laid

L4 larvae were maintained individually on 35 mm NGM plates with *E. coli* OP50 lawns (n = 10 per trial), and transferred every 24 hrs until unfertilised oocyte production ceased, with a minimum of 12 days scored for all worms. For scoring, the open Petri dish was placed on an inverted plate bearing a grid of parallel lines, allowing the plate to be efficiently scored with a series of vertical sweeps. Where clumps of oocytes were seen, these were carefully separated using a worm pick, to count individual oocytes. *P. pacificus* and *P. exspectatus* also laid some dead eggs, consistent with previous observations (R.J. Sommer, personal communication), which were discounted.

### Gel electrophoresis and quantitation of yolk proteins

Quantitation of vented yolk protein was performed as previously described^14^. Briefly, 100–200 L4 larvae were maintained on 35 mm plates with *E. coli* OP50 lawns and transferred daily to new plates. Following transfer, vented yolk was washed off with 1 ml of M9 containing 0.001% NP-40 to solubilise vitellogenin, as described^68^, and 2 μg/ml BSA as an external standard. It was then centrifuged to separate out fractions prior to lyophilisation. After lyophilisation yolk was resuspended in a solution made up of 30 μl 4% SDS solution pH 9, 10 μl DTT, 1 ml 1 M Tris HCL pH 8, 10 μl 0.5 M EDTA, 5 ml Milli-Q water, and 12 mg bromophenol blue.

Quantitation of internal (non-vented) yolk used a protocol similar to that previously described^13^. Briefly, 10 animals per condition per treatment were transferred to NGM plates lacking bacteria and allowed to crawl for 30–60 s to remove bacteria from the nematode surface. They were then transferred to 25 µl M9 and immediately frozen at −80 °C until used.

Samples were mixed with 25 μl of 2× Laemmli sample buffer (Sigma-Aldrich), incubated at 70 °C and vortexed periodically for 15 min, and then incubated at 95 °C for 5 min and centrifuged at 6,000 rpm for 15 min. For all samples, sodium dodecyl sulphate–polyacrylamide gel electrophoresis (SDS-PAGE) was then performed, using Criterion XT Precast Gels 4–12% Bis-Tris (Invitrogen) and XT MOPS (Invitrogen) as a running buffer (7:1 ratio with Milli-Q water) at 90 V. Gels were stained with colloidal Coomassie blue as described^69^, using 5% aluminum sulphate-(14-18)-hydrate and 2% orthophosphoric acid (85%) to create colloidal particles. Gels were analyzed using ImageQuant LAS 4000 (GE Healthcare). Protein band identification was based on published data^70^. Within lanes, vented YPs were normalised to the BSA that had been added during sample collection as an external standard. For samples of internal YPs, YP bands were normalised to myosin as a standard to account for protein loss during gel loading, as well as to allow for normalisation to nematode size when comparing different species.

### Measurement of nematode senescent pathologies

Nomarski microscopy images of senescent pathologies in cohorts of worms (n = 5–10 per time point per condition per trial) were captured on days 1, 4, 7, 11 and 14 and images analysed either quantitatively (intestine, yolk pools), or semi-quantitatively and blind (pharyngeal deterioration, gonad atrophy and fragmentation, uterine tumours) by several trained observers as described^8,9,71^. In brief, intestinal atrophy was quantified by measuring the intestinal width at a point between the posterior gonad and anus, subtracting the width of the intestinal lumen, and dividing by the body width to obtain an estimate of intestinal cross-sectional width normalised to body size. Yolk pool accumulation was measured by dividing the area of yolk pools by the area of the body visible in the field of view at 630x magnification. For pharyngeal deterioration, gonad atrophy and fragmentation and uterine tumours, images were assigned scores of 1–5, where 1 = youthful, healthy appearance; 2 = subtle signs of deterioration; 3 = clearly discernible, mild pathology; 4 = well developed pathology; and 5 = tissue so deteriorated as to be barely recognisable (e.g., gonad completely disintegrated), or reaching a maximal level (e.g. large tumour filling the entire body width).

Scoring was adapted to the different nematode species as follows. Scoring of pharyngeal senescence in *Pristionchus* species took into account the normal absence of a grinder^72^. For scoring of gonadal senescence, the criteria for a healthy gonad included whether the two arms of the gonad touch one another (which they do in young adults). Animals where the gonad arms do not touch, or which show narrowing on the gonad arms were given a score of 2, both features a score of 3, and in addition gonad arm fragmentation a score of 4. For uterine tumour scoring in young unmated females an empty uterus lacking eggs as was given a score of 1.

### Analysis and comparison of pathology progression

In order to prepare data for modelling, data were pre-processed for the following reasons. Peak pathology level for *C. elegans* and most mated animals (which shows maximum pathology progression rate) is mostly reached by day 14, after which pathology levels plateau. Thus, pathology data after day 14 obscures the calculation of gradients representing initial rate of pathology progression. Intestine scores for each combination of species and treatment were normalised to the respective mean intestine score on day 1. Therefore, normalised intestinal pathology scores represent the change in percentage of intestinal volume, accounting for differences in terms of the ratio of intestinal width to whole body width between species. For yolk pool scores no normalisation was used because percentage of the body cavity containing yolk pools was measured. Yolk pools were analysed as score + 1, in order to ensure all data were positive (a requirement for Gamma regression). Generalised linear models (GLMs) were applied to the data to test the effect of species, treatment and trial on pathology progression. Interactions terms between these variables were also included. Different model families and link functions were systematically applied to each pathology dataset according to the type of data recorded. For positive, continuous variables (intestine and yolk pools), pathology scores were modelled as Gaussian and Gamma distributed variables. For ordinal variables (tumour, gonad and pharynx), cumulative link models were applied to the data using the *ordinal* package in R (ref. ^73^).

The following models were selected for each pathology based on minimisation of the Akaine Information Criterion (AIC), meaning that these models explained the most variance in pathology progression using the fewest number of terms: (i) intestine: Gaussian distributed response variable with inverse link function; (ii) yolk pools: Gamma distributed response variable with identity link function; and (iii) tumour, gonad and pharynx: ordinal distributed response variable with log-gamma link function. The vast majority of trial terms were not statistically significant across all pathologies, indicating that differences in pathology progression were reproducible and robust. Hence, trial was not considered further as a variable. In order to compare whether differences in pathology progression between combinations of species and treatment were statistically significant, *t*-tests were applied explicitly to comparisons of interest using the *multcomp* package in R (ref. ^74^).

### Statistics and reproducibility

For all sample images displayed, experiments were repeated a minimum of three times independently and checked to ensure the reproducibility of results.

### Inter-pathology relationships and comparisons to lifespan

In order to compare differences in pathology progression across species and treatments, the gradients from each model were transformed into Z-scores (which describe a value’s relationship to the mean of a group of values), necessary to compare pathologies to one another. The pathology Z-scores are displayed and compared as heatmaps, using pairwise Euclidean differences to cluster pathologies and species/treatments according to profile similarity. In order to model the impact of pathology Z-scores on lifespan, linear regression was performed, using the pathology Z-score as the independent variable and the inverse of mean lifespan as the dependent variable. In order to assess the combined impact of all pathologies on lifespan, the median of the pathology Z-scores was used as the independent variable. The Z-scores for all pathologies were found to be statistically significant, and the median pathology Z-score was found to perform better than the individual pathologies.

### Ablation of the germline using laser microsurgery

Live L1 larvae were mounted on a glass slide on a 5% agar pad with levamisole as anaesthetic. The concentration of levamisole and M9 buffer/Milli-Q water ratio was optimised for each sibling species pair: 0.2 mM levamisole in M9 for *C. elegans, C. inopinata, C. tropicalis* and *C. wallacei*; 0.2 mM levamisole in a 1:1 ratio of M9 to Milli-Q water for *C. briggsae* and *C. nigoni*; and 0.1 mM levamisole in M9 for *P. pacificus* and *P. exspectatus*. Ablations were performed using a Zeiss Axioplan 2 fitted with an Andor MicroPoint laser unit (CE N2 Laser with Ctlr/PSU/IntLk) at 440 nm and Dye Cell 435 nm filter. Germline precursor cells (Z2 and Z3) were identified by morphology and position with Nomarski optics and ablated in newly-hatched L1 animals using a standard protocol^75^.

Following ablation, larvae were transferred to fresh plates by washing them off with M9 buffer (30 μl) for all the pairs of species except for *C. briggsae* and *C. nigoni* which were recovered in a 1:1 ratio of M9 to Milli-Q water (total 30 μl). All ablated animals were then allowed to recover to day 1 of adulthood and checked under a Nikon SMZ645 microscope for both a lack of egg laying, indicating that the ablation was successful, as well as the presence of a vulva, indicating that the Z1 and Z4 cells were intact. For unmated females which do not lay eggs, 15–20 randomly selected worms per condition per trial were checked using Nomarski microscopy (×100 magnification with a ×10 air objective, without anaesthesia) on an NGM plate for the presence of a fully developed gonad, and none were found to have one. Mock treatment animals underwent the same manipulations as ablated animals apart from being shot with the laser microbeam. Ablation of the somatic gonad (by removing the Z1-4 gonad precursor cells) was performed in a similar manner; the resulting adult worms were checked to confirm absence of a vulva.

### Reporting summary

Further information on research design is available in the Nature Portfolio Reporting Summary linked to this article.

## Supplementary information

Supplementary Information

Reporting Summary

## Data Availability

The data generated in this study are provided in the article and its Supplementary Information files. Source data are provided with this paper.

## Source data

Source Data
