# Supplementary material for: C. elegans ageing is accelerated by a self-destructive reproductive programme
Source: Nat Commun. 2023 Jul 20;14:4381. doi: 10.1038/s41467-023-40088-1 (PMC10359416; doi:10.1038/s41467-023-40088-1)
Supplement: Supplementary file 1 — Supplementary Information [file 41467_2023_40088_MOESM1_ESM.pdf]

Supplementary Information

C. elegans ageing is accelerated by a self-destructive reproductive programme

Kern et al. (2022)

Supplementary Figure 1. Greater vitellogenin accumulation and venting in hermaphrodites than females.

Supplementary Figure 2. More severe senescent pathology and shorter lifespan in hermaphroditic species.

Supplementary Figure 3. Degenerative changes in ultrastructure in hermaphrodites undergoing putative reproductive death.

Supplementary Figure 4. Putative reproductive death is constitutive in hermaphrodites and mating-induced in females.

Supplementary Figure 5. Mating induces putative reproductive death and reduces lifespan in females.

Supplementary Figure 6. Germline ablation suppresses severe senescent pathologies in hermaphrodites.

Supplementary Figure 7. Germline ablation greatly extends lifespan in hermaphrodites but not females.

Supplementary Figure 8. Putative reproductive death is promoted by insulin/IGF-1 signalling (IIS).

Supplementary Figure 9. Sequence alignment of *fem-3*.

Supplementary Table 1. Female sibling species are longer lived than hermaphrodites when bacterial pathogenicity is excluded.

Supplementary Table 2. Comparison of hermaphrodite and female sibling species lifespans following mating.

Supplementary Table 3. Comparison of lifespans of feminised and wild-type hermaphrodites, and female sibling species.

Supplementary Table 4. Comparison of lifespans of hermaphrodite and female sibling species following germline ablation.

Supplementary Table 5. Orthologues of *C. elegans fog-2*.

Supplementary Table 6. Orthologues of *C. elegans fem-3*.

Supplementary Table 7. List of primers used for the dsRNA synthesis.

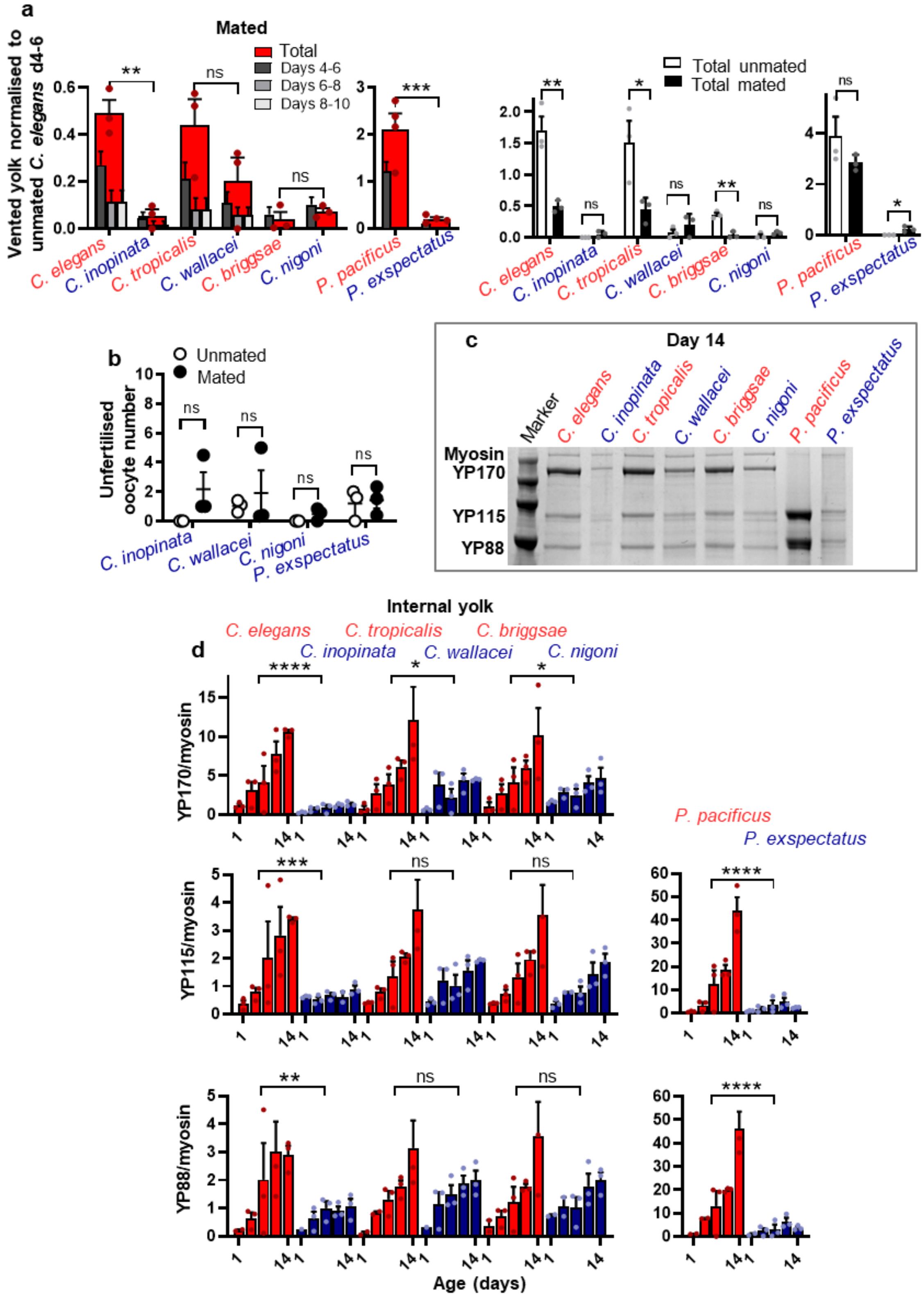

Supplementary Figure 1. **Greater vitellogenin accumulation and venting in hermaphrodites than females. a** Mating reduces venting in hermaphrodites and has little effect on venting by females. Vented YP170 in mated *Caenorhabditis* species and YP115 in mated *Pristionchus*

species. Data normalised to unmated *C. elegans* peak venting period, d4-6 (c.f. Fig. 1b). Mating reduces levels of venting in hermaphrodites, which may reflect vitellogenin uptake by the additional oocytes that become eggs due to increased sperm availability. **b** Mating does not significantly increase unfertilised oocyte production in females. **a**, **b** One-way ANOVA (Bonferroni correction) and one-sample t-test (two-tailed) used based on the number of samples being compared. **c**, **d** Greater internal levels of YP170 in *Caenorhabditis* hermaphrodites, and YP115/YP88 in *P. pacificus* hermaphrodites (*Pristionchus* lack YP170). YP bands normalised to myosin to adjust for species differences in body size. One-way ANCOVA. **a**, **c**, **d** Protein gel electrophoresis data with colloidal Coomassie blue staining. For full gel see Source Data. Mean ± s.e.m. of 3 trials displayed. * p <0.05, ** p <0.01, *** p <0.0001, **** p <0.00001 (p value from left to right = **a left**: 0.0022, 0.1901, 0.3904, 0.0002; **a right**: 0.0057, 0.1321, 0.0416, 0.3310, 0.0020, 0.2028, 0.2458, 0.0223; **b**: 0.2164, 0.8888, 0.9876, 0.9993; **d top**: 0.0000, 0.0133, 0.0133; **d middle**: 0.0006, 0.1316, 0.1316, 0.0000; **d bottom**: 0.0070, 0.2604, 0.2085, 0.0000).

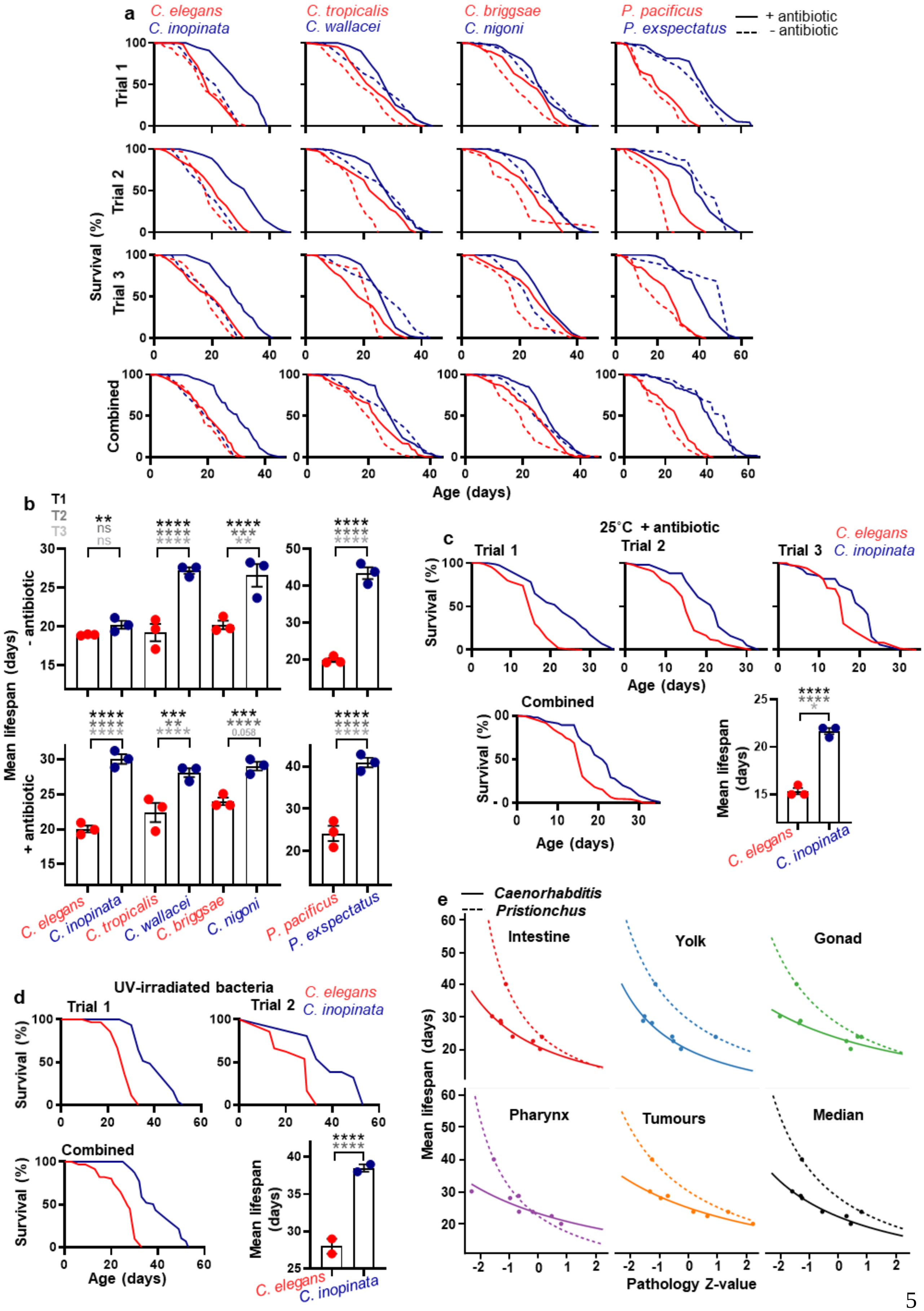
a
C. elegans
C. inopinata
C. tropicalis
C. wallacei
C. briggsae
C. nigoni
P. pacificus
P. exspectatus
+ antibiotic
- antibiotic
Trial 1
Trial 2
Trial 3
Combined
Survival (%)
Age (days)
b
T1
T2
T3
- antibiotic
+ antibiotic
Mean lifespan (days)
c
25°C + antibiotic
Trial 1
Trial 2
Trial 3
Combined
Survival (%)
Age (days)
Mean lifespan (days)
d
UV-irradiated bacteria
Trial 1
Trial 2
Combined
Survival (%)
Age (days)
Mean lifespan (days)
e
Caenorhabditis
Pristionchus
Intestine
Yolk
Gonad
Pharynx
Tumours
Median
Mean lifespan (days)
Pathology Z-value

Supplementary Figure 2. More severe senescent pathology and shorter lifespan in hermaphroditic species. a,b Survival curves from individual and combined trials of sibling species. In the presence of antibiotics, females are longer lived in all cases (n=100 per trial). **c** *C. inopinata* is also longer lived than *C. elegans* at 25˚C (with carbenicillin), a more optimal culture temperature for the former species (n=100 per trial). **d** *C. inopinata* is also longer-lived than *C. elegans* where both are maintained on UV-irradiated *E. coli* (n=100 per trial). **e** Linear regression comparing each individual pathology and median of all pathologies to lifespan. The Z-scores for all pathologies were found to be statistically significant, and the median pathology Z-score was found to perform better than the individual pathologies. This suggests that lifespan is a function of overall pathology level. X axis, pathology Z-score calculated from pathology measurements through time (c.f. Supplemental Fig. 4a). Y axis, mean lifespan. A line of best fit is drawn for *Caenorhabditis* species and based on this a hypothetical line is drawn for the *Pristionchus* species. * p <0.05, ** p <0.01, *** p <0.0001, **** p <0.00001 by Log rank test for pairs of individual trials, and mean ± s.e.m. of 3 trials with each dot representing one trial displayed. For details on statistics and raw lifespan data see Supplemental Table 1 and Source Data File, respectively.

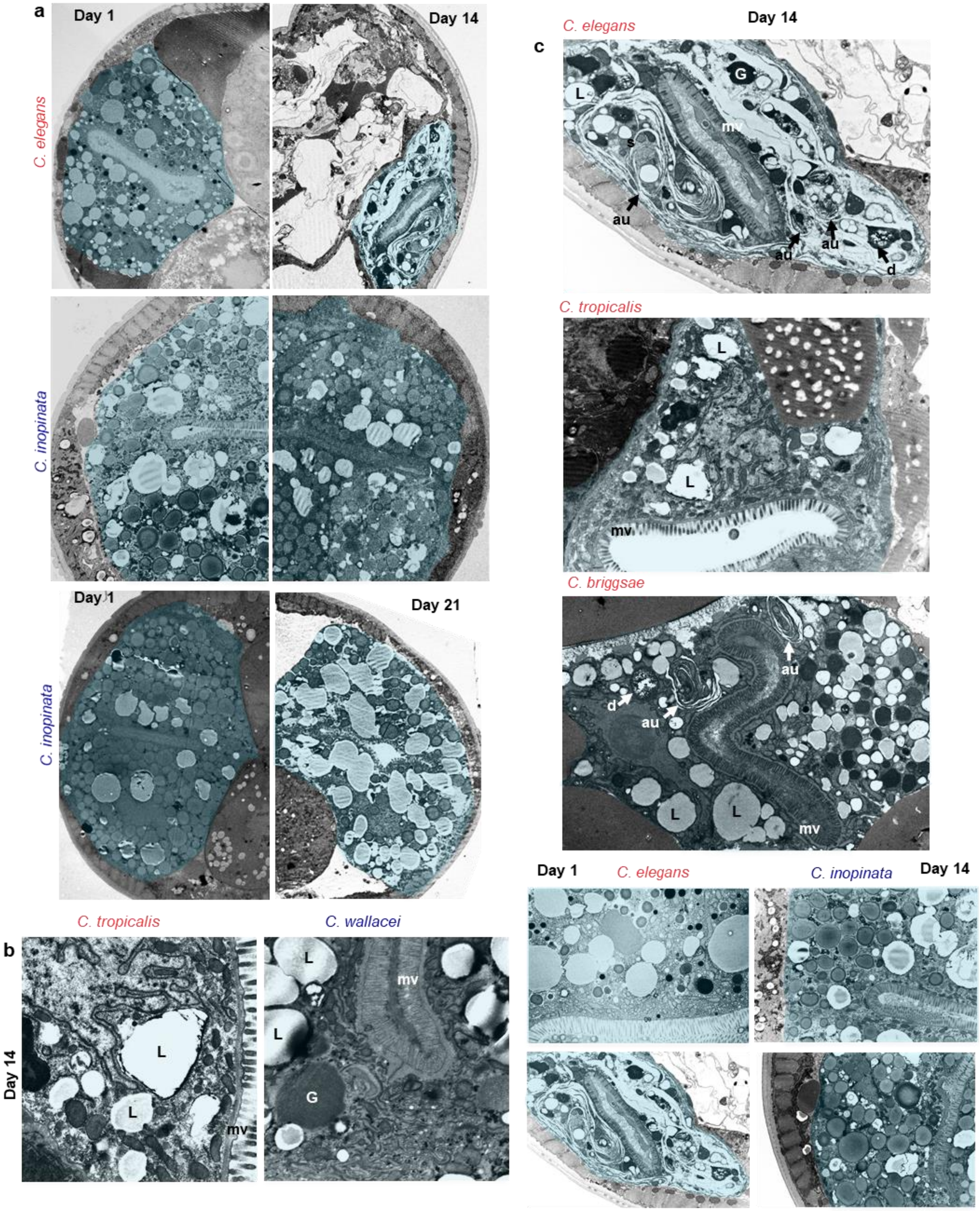

Supplementary Figure 3. **Degenerative changes in ultrastructure in hermaphrodites undergoing putative reproductive death. a** Low magnification representative TEM showing intestinal atrophy in

C. elegans with age and little change in intestinal size in *C. inopinata* even by d21. Halves of nematode sibling species pairs are taken from approximately the same region of the animal (between the posterior end of the posterior uterus and the anus) to avoid the distorting (gut crushing) effects of uterine tumours. Scale 5 µm (5,000x). **b** *C. tropicalis* and *C. wallacei* on d14 showing greater ultrastructural degeneration in the former (c.f. Fig. 2f). Scale 1 µm (20,000x). **c** Hermaphroditic *Caenorhabditis* species showing intestinal degeneration on d14 and comparison to *C. inopinata*. Scale 1 µm (10,000x). Blue, intestine. mv: microvilli; au: autophagosomes; note characteristic multilamellar structure in some cases; L: lipid droplets; G: yolk granules or gut granules (tentative interpretation; gut granules have yet to be clearly identified in TEM studies; D. Hall personal communication).

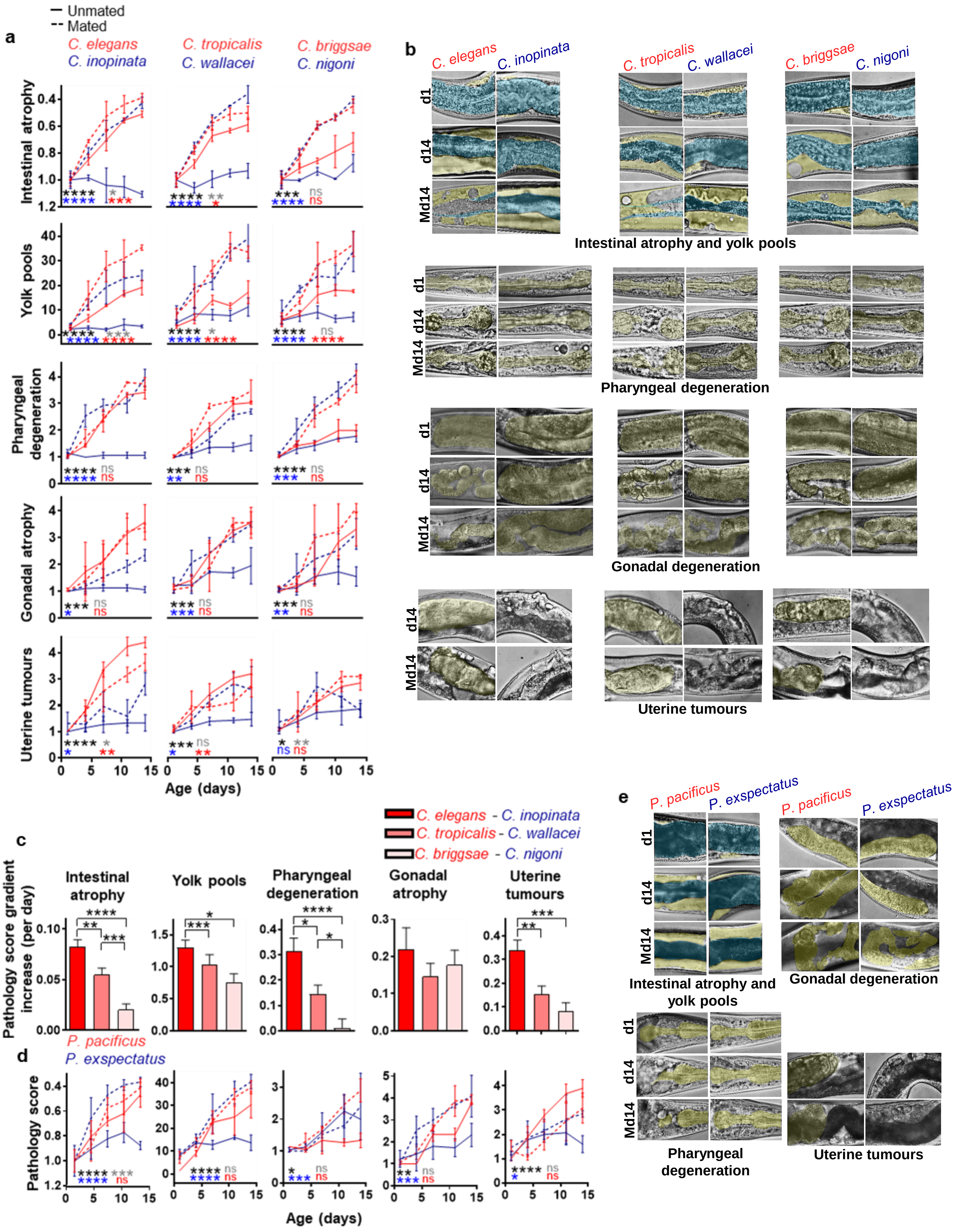

a
Unmated
Mated
C. elegans
C. inopinata
C. tropicalis
C. wallacei
C. briggsae
C. nigoni
Intestinal atrophy
Yolk pools
Pharyngeal degeneration
Gonadal atrophy
Uterine tumours
Age (days)
b
d1
d14
Md14
Intestinal atrophy and yolk pools
Pharyngeal degeneration
Gonadal degeneration
Uterine tumours
c
C. elegans - C. inopinata
C. tropicalis - C. wallacei
C. briggsae - C. nigoni
Pathology score gradient increase (per day)
Intestinal atrophy
Yolk pools
Pharyngeal degeneration
Gonadal atrophy
Uterine tumours
d
P. pacificus
P. exspectatus
Pathology score
Age (days)
e
P. pacificus
P. exspectatus
Intestinal atrophy and yolk pools
Gonadal degeneration
Pharyngeal degeneration
Uterine tumours

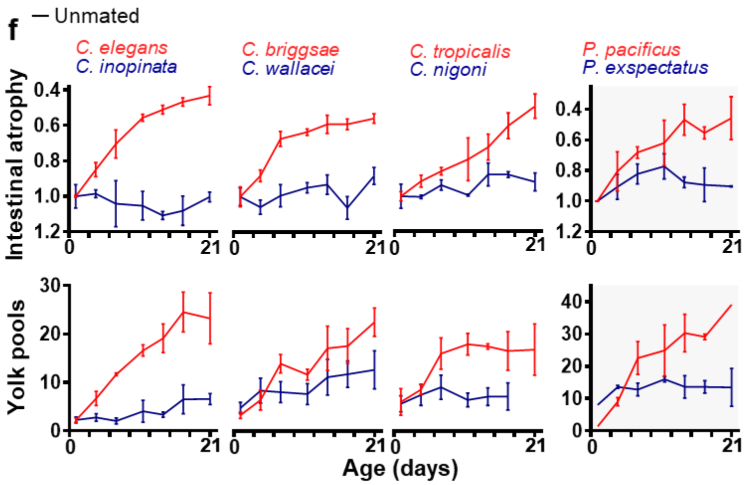

Supplementary Figure 4. **Putative reproductive death is constitutive in hermaphrodites and mating-induced in females. a**, **d** Severe pathology progression in unmated hermaphrodites and mated animals but not virgin females. Tests for statistical significance: black, hermaphrodites vs females; red: mated vs unmated hermaphrodites; blue: mated vs unmated females; grey: mated hermaphrodites vs mated females. Mean ± s.e.m. of 3 trials displayed (n=10 per time point per trial).**b**, **e** Representative Nomarski microscopy images taken for the purpose of scoring/measuring pathologies. Note the position of the gonad around the intestine is different in *Pristionchus* species, with the full gonad arm visible in the same plane only after intestinal atrophy. Yellow: yolk pools, gonad and uterine tumours; blue: intestine. **c** Bar graphs showing difference in gradient between species pairs, defining the following gradient in difference in severity for most pathologies: *C. elegans* - *C. inopinata* > *C. tropicalis* - *C. wallacei* > *C. briggsae* - *C. nigoni*. Subtraction rather than normalisation is used as gradients are already relative values. Mean ± s.t.d. of combined data (n=30 per time point per trial). **f** No increase in intestinal atrophy even up to day 21 in unmated females (c.f. panel a); Mean ± s.e.m. of 3 trials; n=10 per time point per trial. * p <0.05, ** p <0.01, *** p <0.0001, **** p <0.00001 by Cumulative Link Model with gamma link function for pharyngeal degeneration, gonadal degeneration and uterine tumours which are scored ordinals. One-way ANCOVA with score normalised to d1 to determine change in percentage of intestinal mass, and one-way ANCOVA with no normalisation for yolk pools.

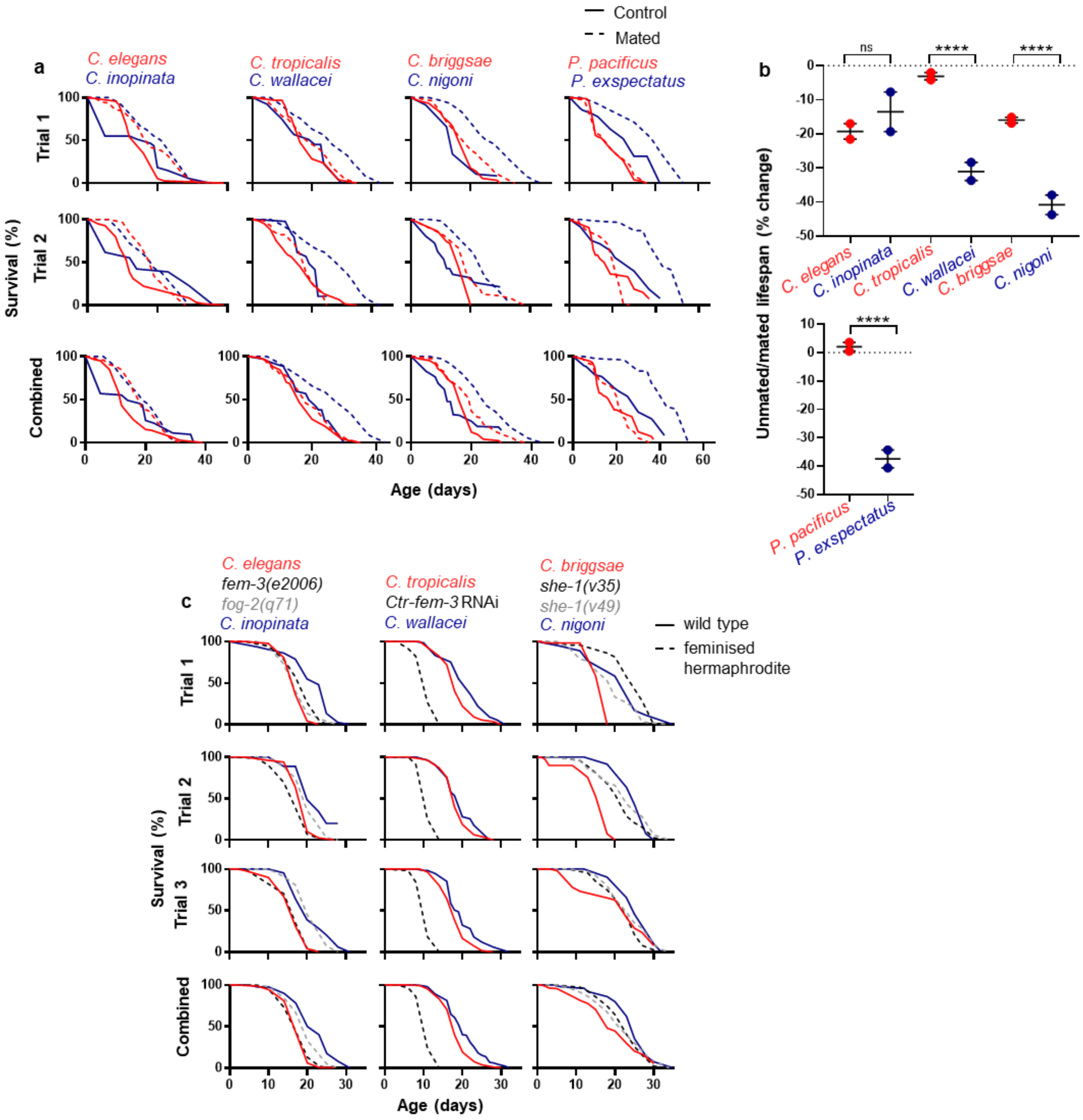

Supplementary Figure 5. **Mating induces putative reproductive death and reduces lifespan in females**. **a** Mating in females significantly reduces lifespan, as previously shown for *C. elegans*[1, 2, 3]. **b** Cox proportional hazard analysis showing greater effect of mating in most females; the lack

of a greater reduction in *C. inopinata* than *C. elegans* could reflect the greater susceptibility of the former to bacterial infection. Mean ± s.e.m. of 2 trials displayed. **c**. Female sibling species are longer lived than spermless hermaphrodites. For details on lifespan statistics and raw lifespan data see Supplemental Table 2 and 3, and Source Data File, respectively. **** p <0.00001.

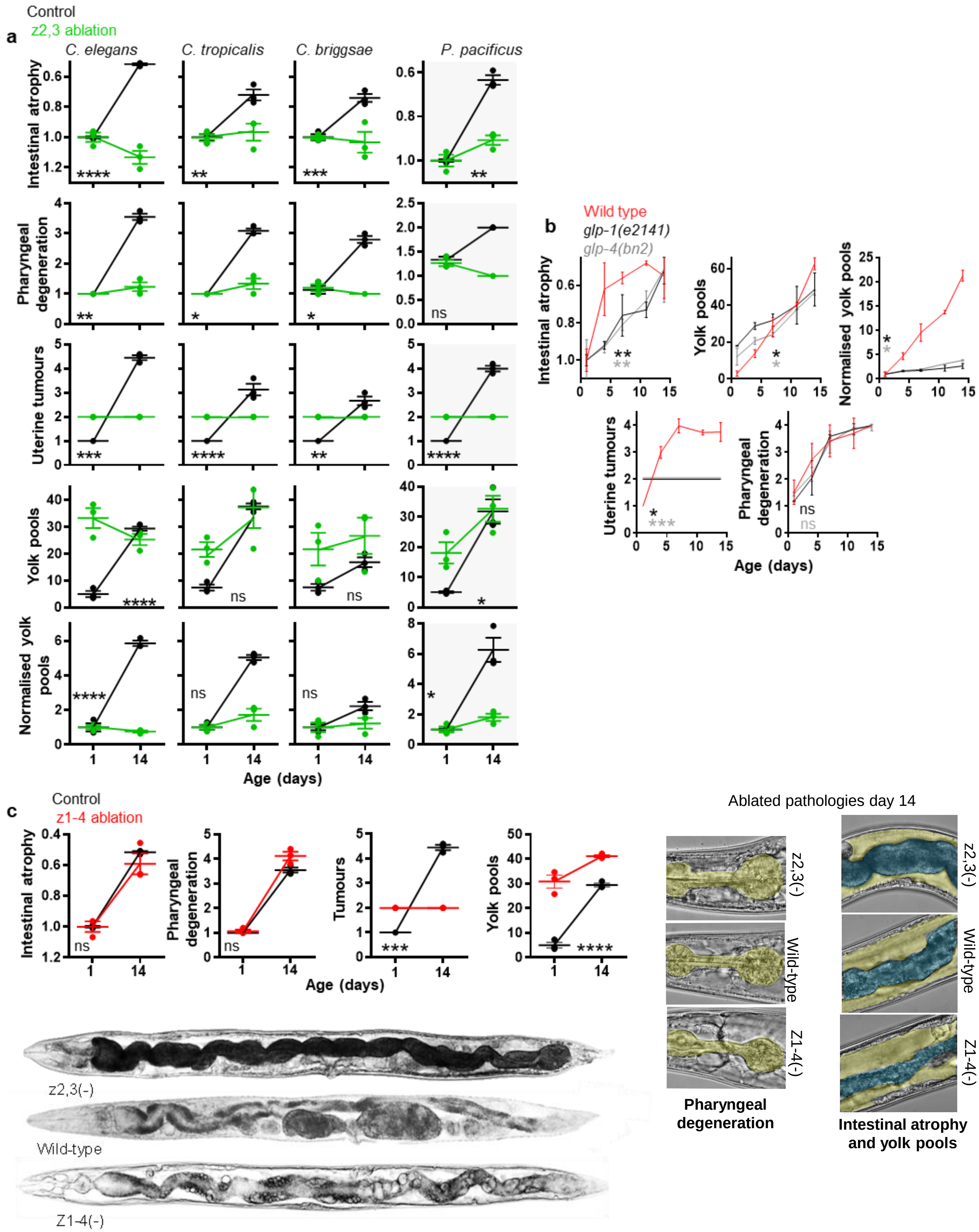

Supplementary Figure 6. **Germline ablation suppresses severe senescent pathologies in hermaphrodites. a** Pathology progression in hermaphrodites is suppressed following z2,3 cell

ablation and germline loss. An exception here is yolk accumulation, which is increased due to the removal of egg laying. **b** Pathology progression in *C. elegans* germline-deficient mutants showing a reduction in most RD-associated pathologies (25˚C). Note that suppression of germline development is not complete in these mutants, in contrast to laser ablated animals, which may account for the weaker suppression of RD-associated pathologies. Mean ± s.e.m. of 3 trials displayed (n=5-10 worms per trial). **c** An intact somatic gonad is required for germline removal to suppress RD-associated pathology. Top left: pathology progression following z1-4 ablation (i.e. full gonad removal) shows mostly no difference to intact animals. The increase in yolk pool size likely reflects absence of yolk efflux due to absence of egg laying and of a vulva. z2,3(-) and z1-4(-) data were collected together with the same control and are comparable (c.f. *C. elegans* data in b). Mean ± s.e.m. of 3 trials displayed (n=5-10 worms per trial). Bottom left: Nomarski images of entire worms, intact (middle) or with germline (top) or whole gonad (bottom) ablated. Note in the germline-ablated animal the dark intestine with no expansion of the gut lumen. The blister-like vesicles in the intestine of whole gonad-ablated worms may be yolk, which is present at high levels due to absence of egg laying and of a vulva. Tumours are absent from ablated animals due to absence of the germline/gonad. The vulva is absent from whole gonad ablated animal, which lack the P cell required for vulval induction. Images have been straightened using the straighten tool in ImageJ. Right: representative Nomarski microscopy images taken for the purpose of scoring pathologies (c.f. Fig. 4b). * p <0.05, ** p <0.01, *** p <0.0001, **** p <0.00001 by Cumulative Link Model with gamma link function for pharyngeal degeneration, gonadal degeneration and uterine tumours which are scored ordinals. One-way ANCOVA with score normalised to d1 to determine change in percentage of intestinal mass, and one-way ANCOVA with no normalisation for yolk pools.

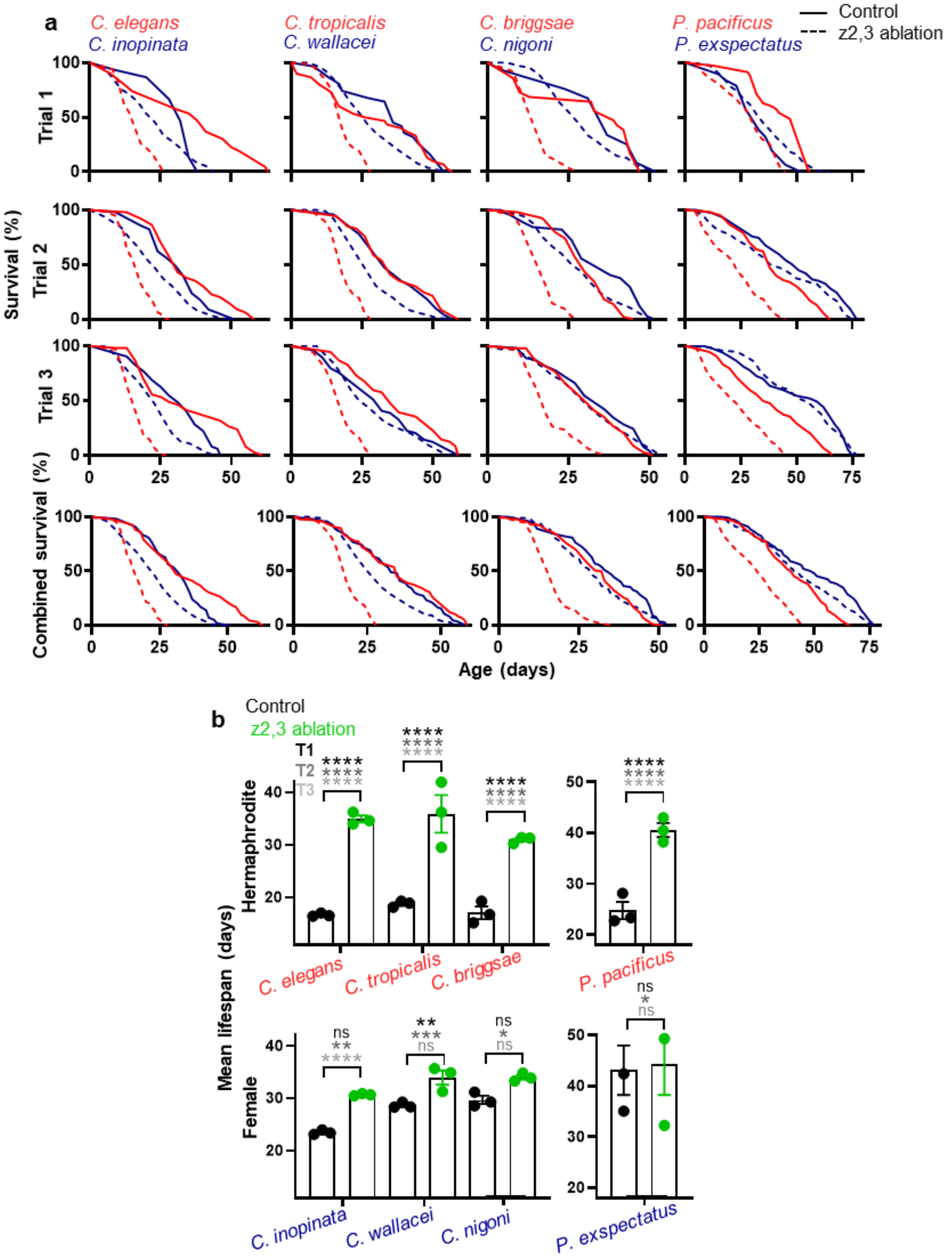

Supplementary Figure 7. **Germline ablation greatly extends lifespan in hermaphrodites but not females. a**, **b** Combined data and individual trial data for lifespans of sibling species after z2,3 ablation. Large effects are seen only in hermaphrodites. Log rank tests for individual trials. Mean ± s.e.m. of 3 trials displayed. * p <0.05, ** p <0.01, *** p <0.0001, **** p <0.00001. For details of statistics and raw lifespan data see Supplemental Table 4 and Source Data File, respectively.

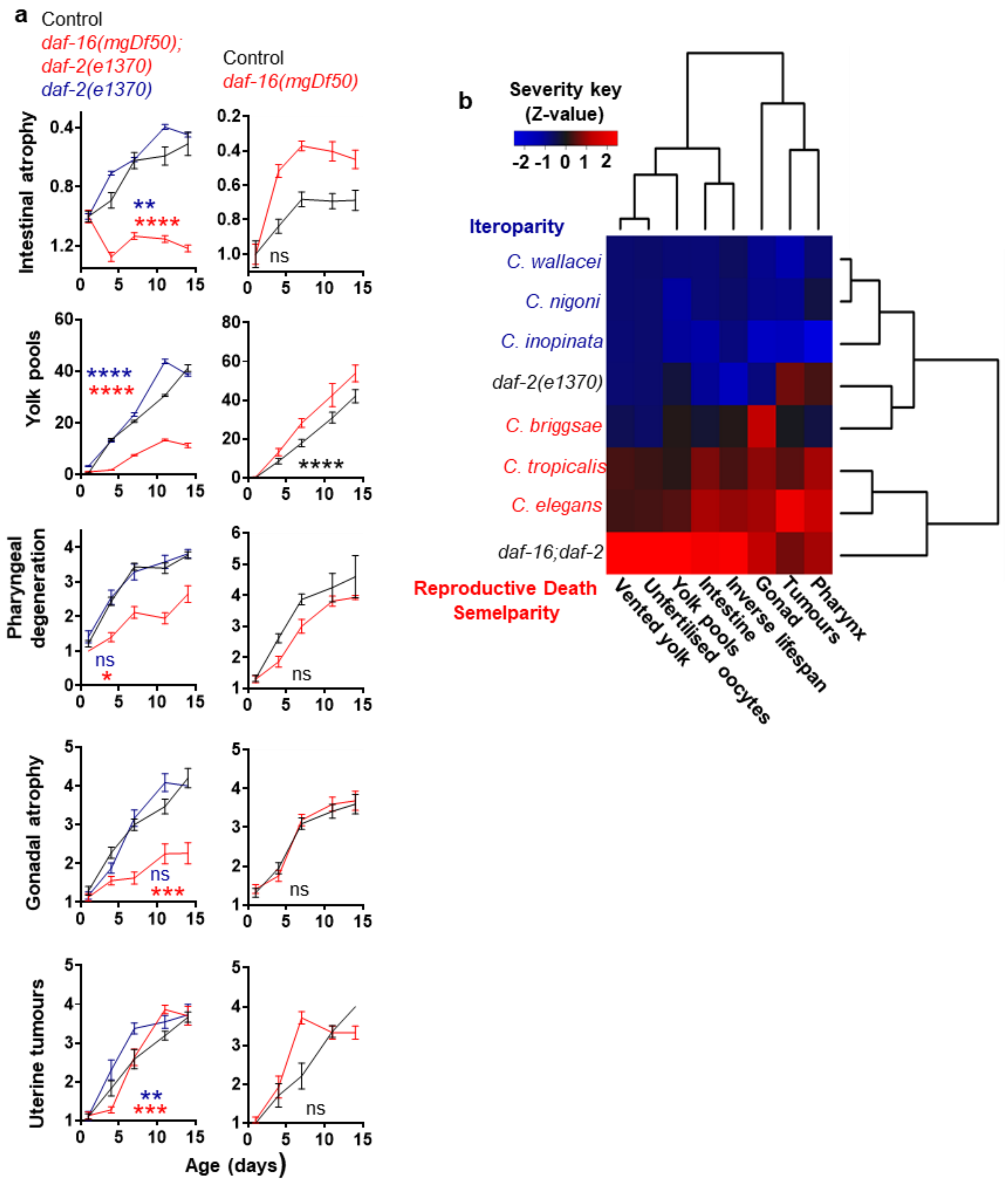

Supplementary Figure 8. **Putative reproductive death is promoted by insulin/IGF-1 signalling (IIS). a** Pathology progression is suppressed in *daf-2(e1370)* mutants, and this is *daf-16* dependant. Mean ± s.e.m. of 3 trials displayed. * *p* <0.05, ** *p* <0.01, *** *p* <0.0001, **** *p* <0.00001 by Cumulative Link Model with gamma link function for pharyngeal degeneration, gonadal degeneration and uterine tumours which are scored ordinals. One-way ANCOVA with score normalised to d1 to determine change in percentage of intestinal mass, and one-way ANCOVA with no normalisation for yolk pools. **b** Lifespan, putative reproductive death-associated pathologies and yolk venting and unfertilised oocyte venting follow a continuum with *daf-2* behaving like unmated females and *daf-16; daf-2* mutants like unmated hermaphrodites. Heat map comparing differences in pathology progression, lifespan, yolk and oocyte venting across species

and treatments by transforming the calculated gradients of pathology progression into Z-scores. Hierarchical clustering based on pair-wise Euclidean distances was used to cluster pathologies and species/treatments according to similarity. Sources: Mutant yolk venting and oocyte data: (ref. 5); mutant lifespan data: (ref. 6).

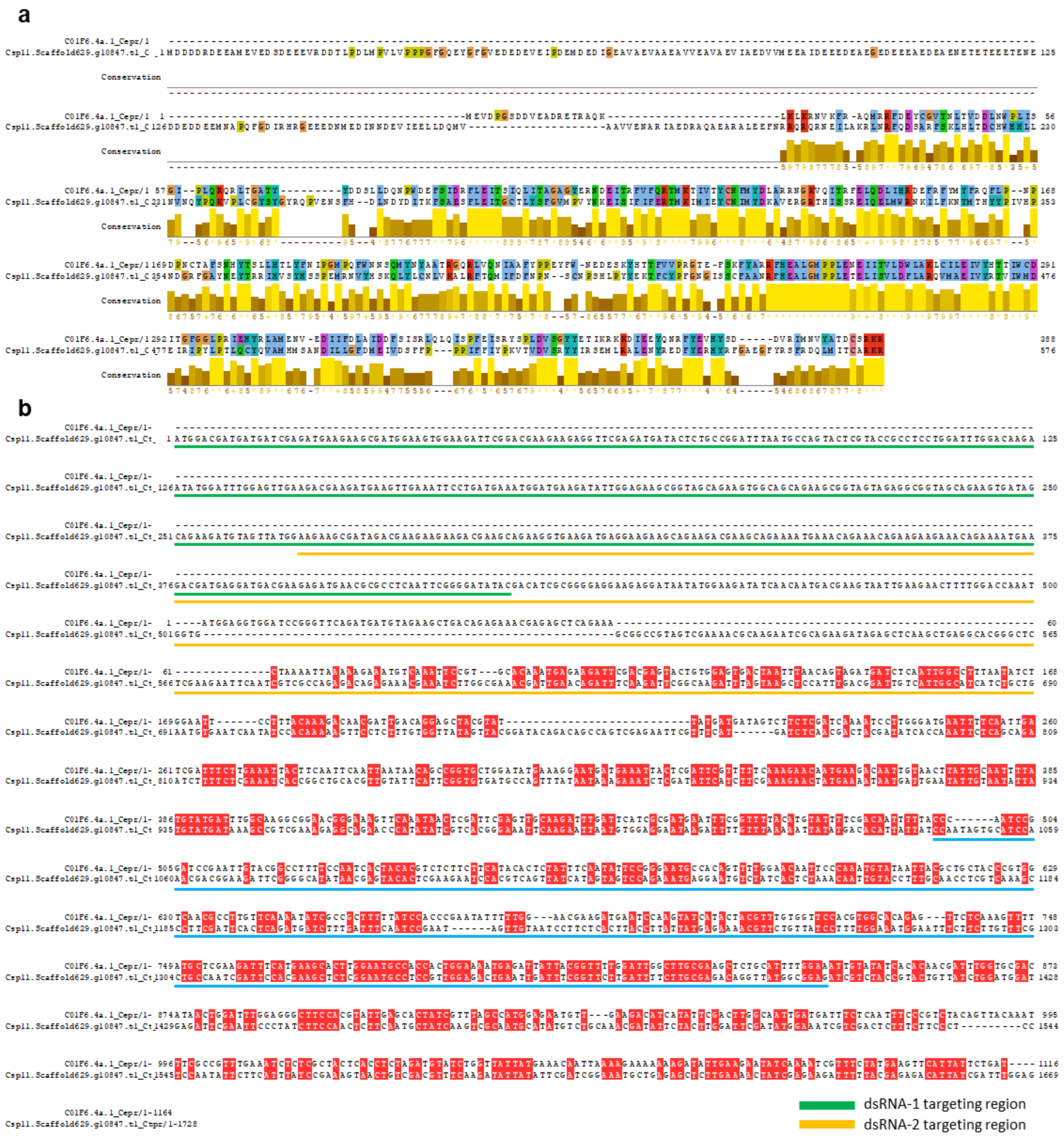

Supplementary Figure 9. **Sequence alignment of *fem-3*.** **a** Protein sequence alignment of *C. elegans fem-3* (C01F6.4a.1) and putative orthologs in *C. tropicalis* (Csp11.Scaffold629.g10847). **b** DNA sequence alignment of *C. elegans fem-3* (C01F6.4a.1) and putative orthologs in *C. tropicalis* (Csp11.Scaffold629.g10847). Three double-stranded RNAs targeting regions are underlined.

Antibiotic and control 20 °C		Number		Lifespan (days)		% change treatment	% change ♀ vs ⚥	Log rank	Cox PH Prob>ChiSq
		death	censored	mean	median				
C. elegans antibiotic	C	219	27	20.14	20.00	6.08	49.92	⚥T vs ⚥ 0.0049	<.0001
	1	55	9	19.93	19.00	5.75	50.82	⚥T vs ⚥ 0.8516	<.0001
	2	92	5	20.92	22.00	10.34	49.12	⚥T vs ⚥ 0.0012	<.0001
	3	72	13	19.25	20.00	1.22	49.75	⚥T vs ⚥ 0.1069	0.0087
C. elegans control	C	216	31	18.99	19.00		6.79	⚥ vs ♀ 0.0057	
	1	112	7	18.85	18.00		12.22	⚥ vs ♀ 0.1287	
	2	54	11	18.96	19.00		2.26	⚥ vs ♀ 0.1192	
	3	50	13	19.02	19.00		6.25	⚥ vs ♀ 0.0491	
C. inopinata antibiotic	C	172	111	30.20	31.00	48.92		♀T vs ♀ < 0.0001	
	1	51	39	30.06	31.00	42.12		♀T vs ♀ < 0.0001	
	2	71	9	31.20	33.00	60.89		♀T vs ♀ < 0.0001	
	3	50	63	28.83	29.00	42.65		♀T vs ♀ < 0.0001	
C. inopinata control	C	130	96	20.28	20.00			⚥T vs ♀T < 0.0001	
	1	41	32	21.15	24.00			⚥T vs ♀T < 0.0001	
	2	43	32	19.39	19.00			⚥T vs ♀T < 0.0001	
	3	46	32	20.21	21.00			⚥T vs ♀T < 0.0001	
C. tropicalis antibiotic	C	264	22	22.55	23.00	15.71	24.75	⚥T vs ⚥ < 0.0001	<.0001
	1	95	10	24.28	23.00	23.20	18.34	⚥T vs ⚥ 0.0003	0.0559
	2	91	5	23.17	25.00	35.46	23.73	⚥T vs ⚥ < 0.0001	<.0001
	3	78	7	19.71	23.00	-5.72	36.14	⚥T vs ⚥ 0.4462	0.0226
C. tropicalis control	C	162	43	19.49	21.00		39.54	⚥ vs ♀ < 0.0001	
	1	78	7	19.71	23.00		33.97	⚥ vs ♀ < 0.0001	
	2	44	16	17.11	18.00		61.59	⚥ vs ♀ < 0.0001	
	3	40	20	20.90	23.00		32.19	⚥ vs ♀ < 0.0001	
C. wallacei antibiotic	C	230	72	28.13	27.00	3.44		♀T vs ♀ 0.1461	
	1	82	25	28.73	28.00	8.82		♀T vs ♀ 0.6742	
	2	78	22	28.67	29.00	3.72		♀T vs ♀ 0.7144	
	3	70	25	26.83	27.00	-2.91		♀T vs ♀ 0.0038	
C. wallacei control	C	240	49	27.19	27.00			⚥T vs ♀T < 0.0001	
	1	87	10	26.40	27.00			⚥T vs ♀T 0.0006	
	2	77	20	27.64	32.00			⚥T vs ♀T 0.0010	
	3	76	19	27.63	32.00			⚥T vs ♀T < 0.0001	
C. briggsae antibiotic	C	273	29	23.91	25.00	20.33	20.52	⚥T vs ⚥ 0.0049	0.0165
	1	107	9	23.44	24.00	18.53	18.92	⚥T vs ⚥ 0.0007	0.0056
	2	86	6	23.43	25.00	9.88	28.01	⚥T vs ⚥ 0.1113	0.8878
	3	80	14	25.07	25.00	28.06	16.33	⚥T vs ⚥ 0.0002	0.629
C. briggsae control	C	190	34	19.87	20.00		35.53	⚥ vs ♀ < 0.0001	
	1	96	20	19.78	24.00		40.80	⚥ vs ♀ < 0.0001	
	2	40	14	21.32	19.00		32.56	⚥ vs ♀ 0.0023	
	3	54	0	19.57	19.00		21.02	⚥ vs ♀ 0.0165	
C. nigoni antibiotic	C	268	49	28.82	29.00	7.00		♀T vs ♀ 0.228	
	1	116	10	27.87	28.00	0.11		♀T vs ♀ 0.5663	
	2	74	21	29.99	29.00	6.11		♀T vs ♀ 0.4993	
	3	78	18	29.16	225.00	23.10		♀T vs ♀ < 0.0001	
C. nigoni control	C	227	46	26.93	25.00			⚥T vs ♀T < 0.0001	
	1	90	11	27.84	28.00			⚥T vs ♀T 0.0004	
	2	76	21	28.26	28.00			⚥T vs ♀T < 0.0001	
	3	61	14	23.69	23.00			⚥T vs ♀T 0.0582	
P. pacificus antibiotic	C	232	26	23.86	27.00	18.71	68.22	⚥T vs ⚥ < 0.0001	<.0001
	1	92	7	20.93	19.00	9.12	95.85	⚥T vs ⚥ 0.1019	0.7063
	2	68	6	27.12	27.00	40.36	43.54	⚥T vs ⚥ < 0.0001	<.0001
	3	72	13	24.59	27.00	17.48	74.23	⚥T vs ⚥ 0.0674	0.018
P. pacificus control	C	274	65	20.10	20.00		120.37	⚥ vs ♀ < 0.0001	
	1	93	12	19.18	20.00		111.05	⚥ vs ♀ < 0.0001	
	2	79	41	19.32	21.00		126.81	⚥ vs ♀ < 0.0001	
	3	102	12	20.93	20.00		119.88	⚥ vs ♀ < 0.0001	
P. exspectatus antibiotic	C	271	46	40.14	41.00	-9.38		♀T vs ♀ 0.0111	
	1	110	11	40.99	42.00	1.26		♀T vs ♀ 0.5208	
	2	81	11	38.92	38.00	-11.17		♀T vs ♀ 0.1355	
	3	105	24	42.85	45.00	-6.91		♀T vs ♀ 0.0017	
P. exspectatus control	C	73	58	44.29	53.00			⚥T vs ♀T < 0.0001	
	1	21	11	40.48	43.00			⚥T vs ♀T < 0.0001	
	2	29	44	43.82	43.00			⚥T vs ♀T < 0.0001	
	3	22	3	46.02	53.00			⚥T vs ♀T < 0.0001	

Antibiotic and control 25 °C		Number		Lifespan (days)		% change ♀ vs ⚥	Log rank	Cox PH Prob>ChiSq
		death	censored	mean	median			
C. elegans	C	268	66	15.57	15.00	-25.76	⚥T vs ♀T < 0.0001	0.0407
	1	110	32	15.09	15.00	-33.72	⚥T vs ♀T < 0.0001	
	2	83	22	15.30	15.00	-27.64	⚥T vs ♀T < 0.0001	
	3	75	12	16.53	16.00	-13.35	⚥T vs ♀T 0.0198	
C. inopinata	C	176	60	20.97	22.00			
	1	75	13	22.76	22.00			
	2	27	27	21.15	22.00			
	3	74	20	19.08	21.00			

UV irradiated bacteria 20 °C		Number		Lifespan (days)		% change ♀ vs ⚥	Log rank	Cox PH Prob>ChiSq
		death	censored	mean	median			
C. elegans	C	46	27	25.65	28.00	-35.14	⚥T vs ♀T < 0.0001	<.0001
	1	24	18	27.54	27.00	-31.01	⚥T vs ♀T < 0.0001	
	2	22	9	23.77	29.00	-40.75	⚥T vs ♀T < 0.0001	
C. inopinata	C	43	27	39.55	35.00			
	1	30	20	39.92	38.00			
	2	13	7	40.12	33.00			

Supplementary Table 1. **Female sibling species are longer lived than hermaphrodites when bacterial pathogenicity is excluded.** C is combined data from all trials, and T is treatment (antibiotic carbenicillin vs control).

Mated vs control 20 °C		Number		lifespan(days)		% change treatment	% change ♀ vs ⚥	Log rank	Cox PH Prob>ChiSq
		death	censored	mean	median				
	C	123	24	15.01	12.00	-20.72	5.58	⚥T vs ⚥ 0.0004	0.8463
C. elegans	1	41	8	15.62	16.00	-17.14	-5.89	⚥T vs ⚥ 0.0017	0.5995
**mated**	2	82	16	14.91	12.00	-21.36	21.82	⚥T vs ⚥ 0.0194	0.1326
	C	166	18	18.93	18.00		7.19	⚥ vs ♀ 0.0455	
C. elegans	1	112	7	18.85	18.00		12.22	⚥ vs ♀ 0.1287	
**control**	2	54	11	18.96	19.00		2.26	⚥ vs ♀ 0.1192	
	C	105	16	15.84	14.00	-21.91		♀T vs ♀ 0.0213	
C. inopinata	1	74	13	14.70	18.00	-30.51		♀T vs ♀ 0.0012	
**mated**	2	31	3	18.17	14.00	-6.31		♀T vs ♀ 0.6203	
	C	84	64	20.29	19.00			⚥T vs ♀T 0.1954	
C. inopinata	1	41	32	21.15	24.00			⚥T vs ♀T 0.6610	
**control**	2	43	32	19.39	19.00			⚥T vs ♀T 0.1117	
	C	144	7	18.07	16.00	-3.54	10.64	⚥T vs ⚥ 0.7607	<.0001
C. tropicalis	1	83	7	19.31	16.00	-2.00	-2.33	⚥T vs ⚥ 0.8952	0.0069
**mated**	2	61	0	16.20	16.00	-4.12	13.16	⚥T vs ⚥ 0.9253	0.0017
	C	123	22	18.73	18.00		44.13	⚥ vs ♀ < 0.0001	
C. tropicalis	1	78	7	19.71	23.00		33.97	⚥ vs ♀ < 0.0001	
**control**	2	45	15	16.89	18.00		63.63	⚥ vs ♀ < 0.0001	
	C	83	55	19.99	19.00	-25.96		♀T vs ♀ < 0.0001	
C. wallacei	1	30	21	18.86	21.00	-28.56		♀T vs ♀ 0.0048	
**mated**	2	53	34	18.33	19.00	-33.69		♀T vs ♀ < 0.0001	
	C	164	30	27.00	27.00			⚥T vs ♀T 0.0441	
C. wallacei	1	87	10	26.40	27.00			⚥T vs ♀T 0.5469	
**control**	2	77	20	27.64	32.00			⚥T vs ♀T 0.0185	
	C	180	1	16.62	16.00	-15.34	-12.04	⚥T vs ⚥ < 0.0001	<.0001
C. briggsae	1	123	0	16.78	16.00	-15.14	-6.17	⚥T vs ⚥ 0.0004	<.0001
**mated**	2	57	1	16.27	16.00	-16.90	-9.41	⚥T vs ⚥ 0.0159	0.1378
	C	150	20	19.63	20.00		33.53	⚥ vs ♀ < 0.0001	
C. briggsae	1	96	20	19.78	20.00		40.86	⚥ vs ♀ < 0.0001	
**control**	2	54	0	19.57	19.00		21.02	⚥ vs ♀ 0.0165	
	C	161	66	14.62	12.00	-44.23		♀T vs ♀ < 0.0001	
C. nigoni	1	43	4	15.74	14.00	-43.48		♀T vs ♀ < 0.0001	
**mated**	2	118	62	14.74	12.00	-37.80		♀T vs ♀ < 0.0001	
	C	151	26	26.22	25.00			⚥T vs ♀T 0.0746	
C. nigoni	1	90	12	27.86	28.00			⚥T vs ♀T 0.1812	
**control**	2	61	14	23.69	23.00			⚥T vs ♀T 0.1748	
	C	132	50	19.68	19.00	0.99	33.85	⚥T vs ⚥ 0.2286	<.0001
P. pacificus	1	59	18	19.30	19.00	0.62	37.82	⚥T vs ⚥ 0.8972	0.0027
**mated**	2	73	32	19.95	19.00	3.27	30.31	⚥T vs ⚥ 0.5063	<.0001
	C	172	53	19.48	21.00		120.08	⚥ vs ♀ < 0.0001	
P. pacificus	1	93	12	19.18	20.00		111.05	⚥ vs ♀ < 0.0001	
**control**	2	79	41	19.32	21.00		126.81	⚥ vs ♀ < 0.0001	
	C	82	32	26.34	30.00	-38.58		♀T vs ♀ < 0.0001	
P. exspectatus	1	39	16	26.60	30.00	-34.29		♀T vs ♀ 0.0019	
**mated**	2	43	16	26.00	30.00	-40.67		♀T vs ♀ < 0.0001	
	C	50	55	42.88	43.00			⚥T vs ♀T < 0.0001	
P. exspectatus	1	21	11	40.48	43.00			⚥T vs ♀T < 0.0001	
**control**	2	29	44	43.82	43.00			⚥T vs ♀T 0.0107	

Supplementary Table 2. **Comparison of hermaphrodite and female sibling species lifespans following mating.** C is combined data from all trials, and T is treatment (mated or unmated).

Spermless (25°C)		Number		lifespan(days)		%change vs ⚥	%change vs ♀	Logrank T vs ⚥	Logrank T vs ♀
		death	censored	mean	median				
C. elegans	C	136	50	17.65	17.00		-15.87		<0.0001
	1	48	14	17.54	17.00		-16.37		<0.0001
	2	40	22	18.91	20.00		-9.84		<0.0001
	3	48	14	16.56	17.00		-21.04		<0.0001
C. elegans fem-3	C	215	6	17.73	17.00	0.47	-15.48	0.6434	<0.0001
	1	74	3	18.85	20.00	6.81	-10.15	0.0054	<0.0001
	2	70	2	18.96	17.00	7.45	-9.61	0.0056	<0.0001
	3	71	1	19.02	17.00	7.78	-9.33	0.5030	<0.0001
C. elegans fog-2	C	191	20	20.23	20.00	14.63	-3.56	<0.0001	0.0002
	1	56	11	17.92	17.00	1.55	-14.57	0.3502	<0.0001
	2	67	5	20.76	20.00	17.65	-1.03	0.0026	0.0213
	3	68	4	21.66	20.00	22.75	3.27	<0.0001	0.9243
C. inopinata	C	160	67	20.98	20.00	18.87		<0.0001	
	1	78	3	21.55	23.00	22.14		<0.0001	
	2	31	47	21.47	20.00	21.66		<0.0001	
	3	51	17	18.12	17.00	2.66		<0.0001	
C. tropicalis	C	158	51	18.64	18.00		-9.09		0.0002
	1	54	17	18.92	18.00		-7.70		0.0032
	2	49	16	18.73	18.00		-8.64		0.5954
	3	55	17	18.26	18.00		-10.94		0.0547
C. tropicalis Ctr-fem-3 RNAi	C	151	30	11.22	11.00	-39.79	-45.27	<0.0001	<0.0001
	1	50	11	11.11	11.00	-40.42	-45.83	<0.0001	<0.0001
	2	52	13	11.49	11.00	-38.33	-43.94	<0.0001	<0.0001
	3	49	6	11.05	11.00	-40.70	-46.09	<0.0001	<0.0001
C. wallacei	C	155	78	20.50	20.00	10.00		0.0002	
	1	59	17	21.16	22.00	13.52		0.0032	
	2	45	32	20.25	20.00	8.67		0.5954	
	3	51	29	20.05	19.00	7.59		0.0547	
C. briggsae	C	62	178	19.56	18.00		-19.98		<0.0001
	1	36	66	16.17	18.00		-33.82		0.0391
	2	38	56	15.06	18.00		-38.36		<0.0001
	3	38	56	20.93	23.00		-14.35		0.0102
C. briggsae she-1(v49)	C	100	105	22.03	23.00	12.64	-9.86	0.0001	0.0038
	1	34	32	19.08	20.00	-2.42	-21.91	0.3707	0.0064
	2	34	40	23.02	23.00	17.72	-5.79	<0.0001	0.6090
	3	32	33	24.02	23.00	22.82	-1.72	0.2499	0.3454
C. briggsae she-1(v35)	C	98	124	22.59	23.00	15.53	-7.55	<0.0001	0.1564
	1	21	51	24.63	25.00	25.94	0.78	0.0004	0.5979
	2	39	38	21.64	23.00	10.65	-11.45	<0.0001	0.0054
	3	38	35	22.47	23.00	14.92	-8.03	0.8084	0.0003
C. nigoni	C	180	38	24.44	25.00	24.96		<0.0001	
	1	61	12	22.10	25.00	13.02		0.0391	
	2	63	16	25.30	25.00	29.37		<0.0001	
	3	56	10	25.90	25.00	32.44		0.0102	

Supplementary Table 3. Comparison of lifespans of feminised and wild-type hermaphrodites, and female sibling species. C is combined data from all trials, and T is treatment (sperm knockdown via mutation or RNAi; comparisons between hermaphrodites and female sibling species are also added under T for simplicity).

z2,3, ablation 20 °C		Number		Lifespan (days)		% change treatment	% change ♀ vs ⚥	Log rank	Cox PH Prob>ChiSq
		death	censored	mean	median				
	C	116	22	34.71	31.00	107.89	-11.83	⚥T vs ⚥ < 0.0001	<.0001
C. elegans	1	28	4	36.23	38.00	120.18	-15.35	⚥T vs ⚥ < 0.0001	<.0001
ablated	2	43	9	34.65	31.00	103.14	-10.76	⚥T vs ⚥ < 0.0001	<.0001
	3	45	9	33.99	28.00	105.28	-10.34	⚥T vs ⚥ < 0.0001	<.0001
	C	282	18	16.70	18.00		40.63	⚥ vs ♀ < 0.0001	
C. elegans	1	100	1	16.45	18.00		40.56	⚥ vs ♀ < 0.0001	
control	2	96	3	17.06	18.00		40.60	⚥ vs ♀ < 0.0001	
	3	86	14	16.56	15.00		41.16	⚥ vs ♀ < 0.0001	
	C	110	14	30.61	32.00	30.34		♀T vs ♀ < 0.0001	
C. inopinata	1	15	0	30.67	32.00	32.60		♀T vs ♀ 0.1410	
ablated	2	45	12	30.92	30.00	28.94		♀T vs ♀ 0.0011	
	3	50	2	30.48	31.00	30.38		♀T vs ♀ < 0.0001	
	C	383	37	23.48	23.00			⚥T vs ♀T 0.0003	
C. inopinata	1	119	16	23.13	23.00			⚥T vs ♀T 0.0202	
control	2	119	6	23.98	23.00			⚥T vs ♀T 0.0436	
	3	145	15	23.38	23.00			⚥T vs ♀T 0.0242	
	C	118	20	35.21	35.00	87.21	-3.92	⚥T vs ⚥ < 0.0001	<.0001
C. tropicalis	1	30	0	29.53	29.50	55.97	18.07	⚥T vs ⚥ < 0.0001	0.0027
ablated	2	38	16	36.26	35.00	87.72	-1.61	⚥T vs ⚥ < 0.0001	<.0001
	3	34	20	41.94	47.00	130.90	-25.45	⚥T vs ⚥ < 0.0001	<.0001
	C	213	41	18.81	18.00		52.46	⚥ vs ♀ < 0.0001	
C. tropicalis	1	70	14	18.93	18.00		49.69	⚥ vs ♀ < 0.0001	
control	2	68	21	19.32	18.00		46.77	⚥ vs ♀ < 0.0001	
	3	75	6	18.16	18.00		61.10	⚥ vs ♀ < 0.0001	
	C	129	1	33.83	33.00	17.98		♀T vs ♀ < 0.0001	
C. wallacei	1	31	0	34.87	36.00	23.03		♀T vs ♀ 0.0057	
ablated	2	49	1	35.68	33.00	25.83		♀T vs ♀ 0.0002	
	3	49	0	31.27	32.00	6.86		♀T vs ♀ 0.3265	
	C	386	30	28.67	27.00			⚥T vs ♀T 0.1631	
C. wallacei	1	137	15	28.34	27.00			⚥T vs ♀T 0.7572	
control	2	122	12	28.35	27.00			⚥T vs ♀T 0.7531	
	3	127	3	29.26	25.50			⚥T vs ♀T 0.0801	
	C	110	22	30.90	32.00	80.13	8.73	⚥T vs ⚥ < 0.0001	<.0001
C. briggsae	1	24	5	31.33	39.00	106.39	11.06	⚥T vs ⚥ < 0.0001	0.0001
ablated	2	49	5	30.26	30.00	80.64	11.99	⚥T vs ⚥ < 0.0001	<.0001
	3	37	12	31.42	30.00	62.70	5.92	⚥T vs ⚥ < 0.0001	<.0001
	C	264	12	17.15	18.00		73.08	⚥ vs ♀ < 0.0001	
C. briggsae	1	88	0	15.18	15.00		92.91	⚥ vs ♀ < 0.0001	
control	2	82	8	16.75	18.00		70.59	⚥ vs ♀ < 0.0001	
	3	94	4	19.31	18.00		61.50	⚥ vs ♀ < 0.0001	
	C	101	16	33.59	34.00	13.15		♀T vs ♀ 0.0152	
C. nigoni	1	12	2	34.80	37.00	18.82		♀T vs ♀ 0.1412	
ablated	2	38	9	33.88	35.00	18.58		♀T vs ♀ 0.0140	
	3	51	5	33.28	33.00	6.70		♀T vs ♀ 0.6898	
	C	371	49	29.69	29.00			⚥T vs ♀T 0.0061	
C. nigoni	1	120	20	29.29	28.00			⚥T vs ♀T 0.9253	
control	2	123	12	28.57	29.00			⚥T vs ♀T 0.0050	
	3	128	17	31.19	32.00			⚥T vs ♀T 0.2840	
	C	138	11	40.23	41.00	64.28	20.50	⚥T vs ⚥ < 0.0001	<.0001
P. pacificus	1	35	0	43.00	47.00	52.86	-24.98	⚥T vs ⚥ < 0.0001	<.0001
ablated	2	54	6	40.49	38.00	78.34	21.82	⚥T vs ⚥ < 0.0001	0.0095
	3	49	5	38.21	35.00	63.55	34.00	⚥T vs ⚥ < 0.0001	<.0001
	C	236	83	24.49	25.00		76.91	⚥ vs ♀ < 0.0001	
P. pacificus	1	53	54	28.13	31.00		24.55	⚥ vs ♀ 0.0002	
control	2	90	18	22.71	23.00		86.68	⚥ vs ♀ < 0.0001	
	3	93	11	23.36	23.00		122.01	⚥ vs ♀ < 0.0001	
	C	169	12	48.47	50.00	11.90		♀T vs ♀ 0.0020	
P. exspectatus	1	17	2	32.26	33.00	-7.93		♀T vs ♀ 0.1298	
ablated	2	74	3	49.33	51.00	16.38		♀T vs ♀ 0.0081	
	3	78	7	51.20	59.00	-1.28		♀T vs ♀ 0.7380	
	C	367	111	43.32	41.00			⚥T vs ♀T < 0.0001	
P. exspectatus	1	125	10	35.04	35.00			⚥T vs ♀T 0.0002	
control	2	105	50	42.39	41.00			⚥T vs ♀T < 0.0001	
	3	137	51	51.87	55.00			⚥T vs ♀T < 0.0001	

Supplementary Table 4. **Comparison of lifespans of hermaphrodite and female sibling species following germline ablation.** C is combined data from all trials, and T is treatment (z2,3 ablation vs control).

Species	Type	Stable ID & gene name	Location	Target %id	Query %id
Caenorhabditis brenneri (PRJNA20035)	Many-to-many	WBGene00141755CBN03030 F-box domain-containing protein [Source: UniProtKB/TrEMBL; acc: G0NR54]	Cbre_Contig144:93,719-94,969:1	17.8042	18.3486
Caenorhabditis brenneri (PRJNA20035)	Many-to-many	WBGene00219468CBN32940 (No description)	Cbre_Contig13:62,548-63,612:-1	17.2308	17.1254
Caenorhabditis remanei (PRJNA53967)	1-to-many	WBGene00065932CRE15688 F-box domain-containing protein [Source: UniProtKB/TrEMBL; acc: E3N872]	Crem_Contig147:125,909-127,086:-1	14.4044	15.9021
Caenorhabditis remanei (PRJNA577507 - PX506)	1-to-many	GCK72_021717F-box domain-containing protein [Source: UniProtKB/TrEMBL; acc: A0A6A5GIX5]	V:20,964,520-20,965,697:-1	13.5734	14.9847
Caenorhabditis sinica (PRJNA194557)	1-to-many	Csp5_scaffold_00956.g17079 (No description)	Csp5_scaffold_00956:15,646-18,094:1	14.2857	17.4312
Caenorhabditis zanzibari (PRJEB12596 - JU2190)	Many-to-many	CSP26.g14665 (No description)	CSP26.scaffold01695:21,907-29,411:-1	16.7901	20.7951
Caenorhabditis zanzibari (PRJEB12596 - JU2190)	Many-to-many	CSP26.g14674 (No description)	CSP26.scaffold01695:78,616-81,353:-1	15.6566	18.9602
Caenorhabditis zanzibari (PRJEB12596 - JU2190)	Many-to-many	CSP26.g15834 (No description)	CSP26.scaffold01921:12,710-14,290:-1	17.4174	17.737
Caenorhabditis zanzibari (PRJEB12596 - JU2190)	Many-to-many	CSP26.g15837 (No description)	CSP26.scaffold01921:22,754-24,324:-1	15.8568	18.9602
Caenorhabditis zanzibari (PRJEB12596 - JU2190)	Many-to-many	CSP26.g15840 (No description)	CSP26.scaffold01921:29,231-30,820:-1	14.7132	18.0428
Caenorhabditis zanzibari (PRJEB12596 - JU2190)	Many-to-many	CSP26.g15842 (No description)	CSP26.scaffold01921:33,394-35,889:-1	14.6699	18.3486
Caenorhabditis zanzibari (PRJEB12596 - JU2190)	Many-to-many	CSP26.g15846 (No description)	CSP26.scaffold01921:46,049-48,140:1	15.0121	18.9602
Caenorhabditis zanzibari (PRJEB12596 - JU2190)	Many-to-many	CSP26.g18235 (No description)	CSP26.scaffold02352:1,219-3,859:-1	17.7843	18.6544
Caenorhabditis zanzibari (PRJEB12596 - JU2190)	Many-to-many	CSP26.g21536 (No description)	CSP26.scaffold03017:1,263-2,940:1	17.5127	21.1009
Caenorhabditis zanzibari (PRJEB12596 - JU2190)	Many-to-many	CSP26.g8985 (No description)	CSP26.scaffold00585:15,224-18,508:-1	17.1795	20.4893

Supplementary Table 5. **Orthologues of *C. elegans fog-2*.** List from Wormbase Parasite Version WBPS17 identify 15 orthologues of *C. elegans fog-2* (*Y113G7B.5*).

Species	Type	Stable ID & gene name	Location	Target %id	Query %id
Caenorhabditis zanzibari (PRJEB12596 - JU2190)	1-to-1	CSP26.g3 (No description)	CSP26.scaffold00001:17,381-22,959:-1	33.9713	36.5979
Caenorhabditis briggsae (PRJNA10731)	1-to-1	WBGene00000329Cbr-fem-3 Sex-determination protein fem-3 [Source: UniProtKB/Swiss-Prot; acc: Q8I8U6]	IV:4,390,949-4,394,091:-1	34.4743	36.3402
Caenorhabditis brenneri (PRJNA20035)	1-to-1	WBGene00143028Cbn-fem-3 (No description)	Cbre_Contig125:108,075-110,499:-1	32.4826	36.0825
Caenorhabditis sinica (PRJNA194557)	1-to-1	Csp5_scaffold_00790.g15495 (No description)	Csp5_scaffold_00790:22,851-25,534:-1	34.414	35.567
Caenorhabditis tribulationis (PRJEB12608 - JU2818)	1-to-1	CSP40.g12744 (No description)	CSP40.scaffold01150:122,759-125,524:-1	32.8571	35.567
Caenorhabditis latens (PRJNA248912)	1-to-1	FL83_16550 (No description)	scaffold_152:68,487-71,096:1	28.6713	31.701
Caenorhabditis nigoni (PRJNA384657 - JU1422)	1-to-1	Cni-fem-3Cni-fem-3 (No description)	CM008512.1:5,362,966-5,367,104:-1	34.072	31.701
Caenorhabditis remanei (PRJNA53967)	1-to-many	WBGene00063901Cre-fem-3.2 Sex determination protein FEM-3 [Source: UniProtKB/TrEMBL; acc: Q563Y6]	Crem_Contig62:72,510-74,397:1	27.5395	31.4433
Caenorhabditis sulstoni (PRJEB12601 - JU2788)	1-to-1	CSP32.g12353 (No description)	CSP32.scaffold01101:85,534-87,080:-1	31.6754	31.1856
Caenorhabditis remanei (PRJNA53967)	1-to-many	WBGene00073662Cre-fem-3.1 CRE-FEM-3 protein [Source: UniProtKB/TrEMBL; acc: E3NMC5]	Crem_Contig611:3,689-6,246:-1	26.9663	30.9278
Caenorhabditis tropicalis (PRJNA53597)	1-to-1	Csp11.Scaffold629.g10847 (No description)	Scaffold629:12,602,460-12,605,203:-1	20.6597	30.6701
Caenorhabditis panamensis (PRJEB28259)	1-to-1	CSP28.g19245 (No description)	CSP28.scaffold94_cov83:50,508-52,114:1	23.6702	22.9381
Caenorhabditis becei (PRJEB28243 - QG2083)	1-to-1	CSP29.g4040 (No description)	CSP29.scaffold2337_cov175:610,048-611,842:-1	20.4852	19.5876
Caenorhabditis waitukubuli (PRJEB12602 - NIC564)	1-to-many	CSP39.g24706 (No description)	CSP39.scaffold16213:1,639-3,259:1	19.5652	18.5567
Caenorhabditis waitukubuli (PRJEB12602 - NIC564)	1-to-many	CSP39.g13099 (No description)	CSP39.scaffold05322:627-1,736:1	15.5462	9.53608
Caenorhabditis japonica (PRJNA12591)	1-to-1	WBGene00210371Cjp-fem-3 (No description)	Cjap.Contig18057:7,965-13,175:1	10.4046	9.27835
Caenorhabditis waitukubuli (PRJEB12602 - NIC564)	1-to-many	CSP39.g28022 (No description)	CSP39.scaffold19127:1-746:-1	17.5573	5.92783

Supplementary Table 6. Orthologues of *C. elegans fem-3*. Listing from Wormbase Parasite Version WBPS17 of 17 orthologues of *C. elegans fem-3* (*C01F6.4a.1*), including *Caenorhabditis tropicalis* Csp11.Scaffold629.g10847 (highlighted in green).

dsRNA-1	
Forward: 5'- TAATACGACTCACTATAGGGcgaagaagaagacgaagcag -3'	
Reverse: 5'- TAATACGACTCACTATAGGGcacattcagcagatgatgcc -3'	
dsRNA-2	
Forward: 5'- TAATACGACTCACTATAGGGatggacgatgatgatcga -3'	
Reverse: 5'- TAATACGACTCACTATAGGGcgtatatccccgaattgagg -3'	
dsRNA-3	
Forward: 5'- TAATACGACTCACTATAGGGccaatagtgcatccaaacga -3'	
Reverse: 5'- TAATACGACTCACTATAGGGctccgccataacctgtct -3'	

Supplementary Table 7. List of primers used for dsRNA synthesis. The T7 promoter sequence is added to the 5' ends.

References

1. Gems D, Riddle DL. Longevity in *Caenorhabditis elegans* reduced by mating but not gamete production. *Nature* **379**, 723-725 (1996).

2. Shi C, Murphy CT. Mating induces shrinking and death in *Caenorhabditis* mothers. *Science* **343**, 536-540 (2014).

3. Maures T, Booth L, Benayoun B, Izrayelit Y, Schroeder F, Brunet A. Males shorten the life span of *C. elegans* hermaphrodites via secreted compounds. *Science* **343**, 541-544 (2014).

4. Kern CC, *et al. C. elegans* feed yolk to their young in a form of primitive lactation. *Nat Commun* **12**, 5801 (2021).

5. Bansal A, *et al.* Transcriptional regulation of *Caenorhabditis elegans* FOXO/DAF-16 modulates lifespan. *Longev Healthspan* **3**, 5 (2014).
