# Supplementary figures and images for: C. elegans ageing is accelerated by a self-destructive reproductive programme
Source: Nat Commun. 2023 Jul 20;14:4381. doi: 10.1038/s41467-023-40088-1 (PMC10359416; doi:10.1038/s41467-023-40088-1)

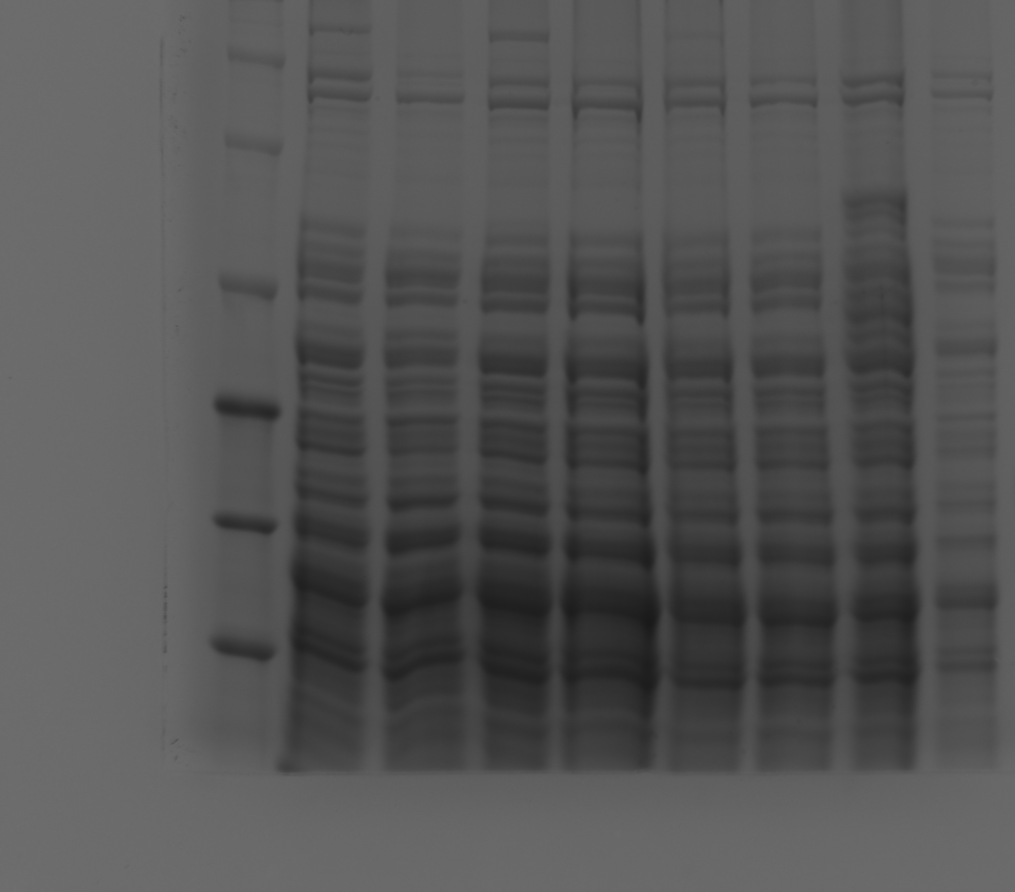

Supplement: Supplementary file 3 — Source Data [file 41467_2023_40088_MOESM3_ESM.zip › Source data file/Gels/Figure 1b gel.tif]

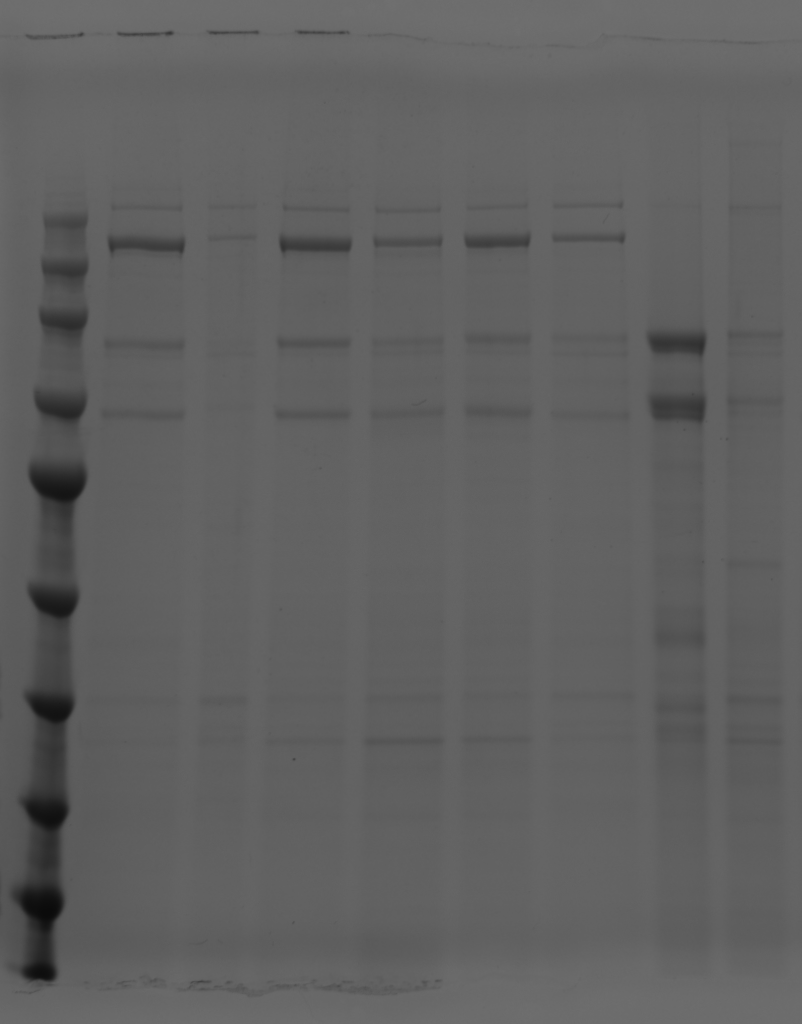

Supplement: Supplementary file 3 — Source Data [file 41467_2023_40088_MOESM3_ESM.zip › Source data file/Gels/Supplementary Figure 1c gel.tif]
